# Steric repulsion counteracts ER–to–lipid droplet protein movement

**DOI:** 10.1126/sciadv.adu6998

**Published:** 2025-09-24

**Authors:** Alicia Damm, Mohyeddine Omrane, Ozren Stojanović, Bianca M. Esch, Mehdi Zouiouich, Maxime Carpentier, Robin Klemm, Florian Fröhlich, Lionel Forêt, Abdou Rachid Thiam

**Affiliations:** ^1^ Laboratoire de Physique de l’École Normale Supérieure (ENS), Université PSL, CNRS, Sorbonne Université, Université de Paris, 75005 Paris, France.; ^2^ Department of Physiology, Anatomy, and Genetics, University of Oxford, Oxford OX13PT, UK.; ^3^ Bioanalytical Chemistry Section, Department of Biology-Chemistry, Osnabrück University, Barbarastrasse 13, 49076 Osnabrück, Germany.; ^4^ Center for Cellular Nanoanalytic Osnabrück (CellNanOs), Osnabrück University, Barbarastrasse 11, 49076 Osnabrück, Germany.

## Abstract

Lipid droplets (LDs) are organelles with a neutral lipid core surrounded by a phospholipid monolayer, which is continuous with the cytoplasmic leaflet of the endoplasmic reticulum (ER). LD function depends on a highly dynamic LD surface proteome. Key proteins continuously exchange between the ER and LDs; however, the mechanisms governing the interorganelle movement and accumulation on the LD surface remain poorly understood. Here, we developed an ex cellulo tool introducing a classification of ER-derived proteins based on their different affinity for LDs. We find that proteins with higher LD affinity can effectively displace those with lower affinity from the LD surface, identifying steric hindrance as a key mechanism in regulating ER-to-LD protein transfer. Consistent with this model, we show that, during adipocyte differentiation Plin1—an adipocyte-specific high-affinity LD protein—reduces the recruitment of ER proteins with lower affinity by displacing them from the LD surface. These findings highlight lateral protein-protein exclusion as a fundamental mechanism in shaping the LD proteome.

## Teaser

Steric hindrance affects the relocation of ER-to-LD proteins and primarily regulates the LD proteome.

## INTRODUCTION

Lipid droplets (LDs) are dynamic organelles central to cellular lipid metabolism, with critical roles beyond lipid storage, including functions in stress responses, immunity, infection control, and cellular development (*1*–*4*). LDs originate from the endoplasmic reticulum (ER), where neutral lipids accumulate within the ER’s membrane bilayer. This accumulation nucleates a neutral lipid “lens” that grows and eventually buds into the cytosol (*5*, *6*). The core of LDs consists of stored neutral lipids, primarily triacylglycerols (TAGs) and cholesterol esters (CEs). Unlike most organelles, which are enclosed by lipid bilayers, LDs are surrounded by a phospholipid monolayer due to their unique oil-phase core (*7*). This structure forms intracellular emulsion droplets with high interfacial tension (*8*), contrasting with the low surface tension of bilayer membranes. The high surface tension makes LDs conducive to recruiting amphipathic molecules, such as proteins, to their surface (*8*–*10*).

Because of their unique structure, LDs’ surface is targeted by peripheral proteins, which are key to most LD functions (*11*). Many of these proteins are cytosolic (class II) and localize to LDs through the CYTOLD pathway (for cytosol-to-LD) via amphipathic helices, lipid anchors, or adaptor proteins (*9*, *12*–*16*). Other proteins (class I) move to the LD from the ER using the ERTOLD pathway (for ER-to-LD), typically having monotopic domains that allow diffusion between the ER and LD surfaces through their physical continuity (*12*, *13*, *17*–*21*). This includes lipid biosynthesis enzymes like acyl–coenzyme A (CoA) synthetase 3 (ACSL3), which generates acyl-CoA for TAG synthesis (*22*), and diacylglycerol O-acyltransferase 2 (DGAT2), which converts diacylglycerol into TAG (*23*). In addition, proteins such as Caveolin2, which are involved in caveolae formation, target newly formed LDs from the ER (*24*, *25*). We recently identified that monotopic proteins may also target LDs from other bilayer compartments, such as late endosomes (*26*). However, despite recent progress, the mechanisms by which monotopic proteins transfer from a phospholipid bilayer to the LD monolayer remain to be better understood (*17*, *18*, *21*, *27*–*29*).

Seipin is an integral ER protein that orchestrates LD formation. It assembles into an oligomeric ring–like structure and interacts with other monotopic proteins like LDAF1 at its center (*30*–*33*). Together, they form the LD assembly complex, which nucleates LDs and facilitates their growth (*30*–*33*). As LDs form, LDAF1 relocates from the seipin complex to the monolayer of the nascent LD (*33*, *34*). Similarly, ACSL3 targets early-stage LDs (*22*, *35*), but not all monotopic proteins follow this pathway. For instance, in *Drosophila* S2 cells, glycerol-3-phosphate acyltransferase 4 (GPAT4), which catalyzes the first step in TAG synthesis, is excluded from nascent LDs and targets mature ones (*36*, *37*). Upon seipin deletion, GPAT4 rapidly targets nascent LDs (*38*), indicating that seipin regulates the ER-to-LD protein movement. This has also recently been evidenced by a single-molecule approach showing that the model LiveDrop peptide [*dm*GPAT4 hairpin motif (*21*)] bidirectionally navigates between the ER and LD via a seipin-controlled ER-LD bridge (*27*).

The CYTOLD pathway has been studied more extensively than the ERTOLD pathway, which remains far less understood. However, recent advances, particularly in *Drosophila* cells, have revealed a few mechanisms that regulate the ERTOLD pathway (*39*). An early ERTOLD pathway, dependent on seipin, governs the initial targeting of proteins from the ER to nascent LDs. In contrast, a late ERTOLD pathway operates independently of seipin (*28*) and is promoted by the hemifusion and physical contiguity between the ER and LDs (*37*, *40*). The functional importance of separate ERTOLD pathways, the selectivity of monotopic proteins targeted to the LD through them, and the driving forces behind the movement of these different ERTOLD cargo proteins to the LDs remain largely unclear.

Evidence suggests that proteins have domains by which they sense the unique physical and chemical properties of LDs. In vitro studies show, in the case of TAG, that hydrophobic motifs favor the LD monolayer (*17*) and that higher phosphatidylcholine (PC) to phosphatidylethanolamine (PE) ratios lower the energy required for protein partitioning to LDs, likely due to the greater lipid packing defects of monolayers (*41*). The type of neutral lipid within LDs also appears to affect protein recruitment in some cases (*9*, *42*). Computational and mutational studies reveal that specific residues, like tryptophan and positively charged amino acids, are crucial for ER-to-LD relocation, as seen in the LiveDrop motif. Conformational changes also regulate protein recruitment, exemplified by UBXD8 and PLIN1 (*18*, *29*, *43*), which use membrane anchoring motifs for LD association from the ER. For instance, PLIN1’s C-terminal amphipathic helix bundle likely “unzips” to bind the LD surface (*44*).

These findings underscore the importance of lipid-membrane/protein interactions in shaping the energy landscape for protein localization to the LD monolayer versus the ER bilayer. Despite these insights, a key question remains: What factors regulate the monotopic protein partitioning level between the ER and LDs and monitor the proteome composition? Addressing these questions is crucial for understanding the occurrence and kinetics of biochemical reactions occurring at the LD surface, which underpins LD biology.

The ERTOLD pathway has primarily been studied in the context of individual proteins, focusing on structural analysis, although free energy differences must determine how much they relocate from the ER to the LD surface. A comparative study of protein enrichment levels and mechanisms at LDs is still lacking. To address this, we developed an ex cellulo tool that quantifies the ER-to-LD partitioning affinity of monotopic proteins. This approach revealed that lateral protein-protein exclusion is critical in controlling protein relocation from the ER to LDs.

## RESULTS

### Proteins target LDs from the ER with a wide range of affinities

We asked to what extent different proteins and motifs partition between ER and LDs. To do so, we picked a subset of ER proteins with different binding motifs and involved in diverse LD functions. These included LDAF1, DGAT2, AGPAT3, PLIN1, CAV2, GPAT4, FAR1, HSD17B13, ACSL3, and some of their hairpin motifs hpDGAT2, hpAGPAT3, and hpGPAT4 (*21*). We included DGAT1, an ER-resident multipass transmembrane protein, as a control. For comparison, we also considered the model membrane anchors HPos and HNeu (*22*), previously developed to investigate LD targeting (*22*). In addition, we examined the behavior of proteins from different organisms that target LDs, such as ERG6 (*Saccharomyces cerevisiae*) and CG2254 (*Drosophila melanogaster*).

**Fig. 1.**
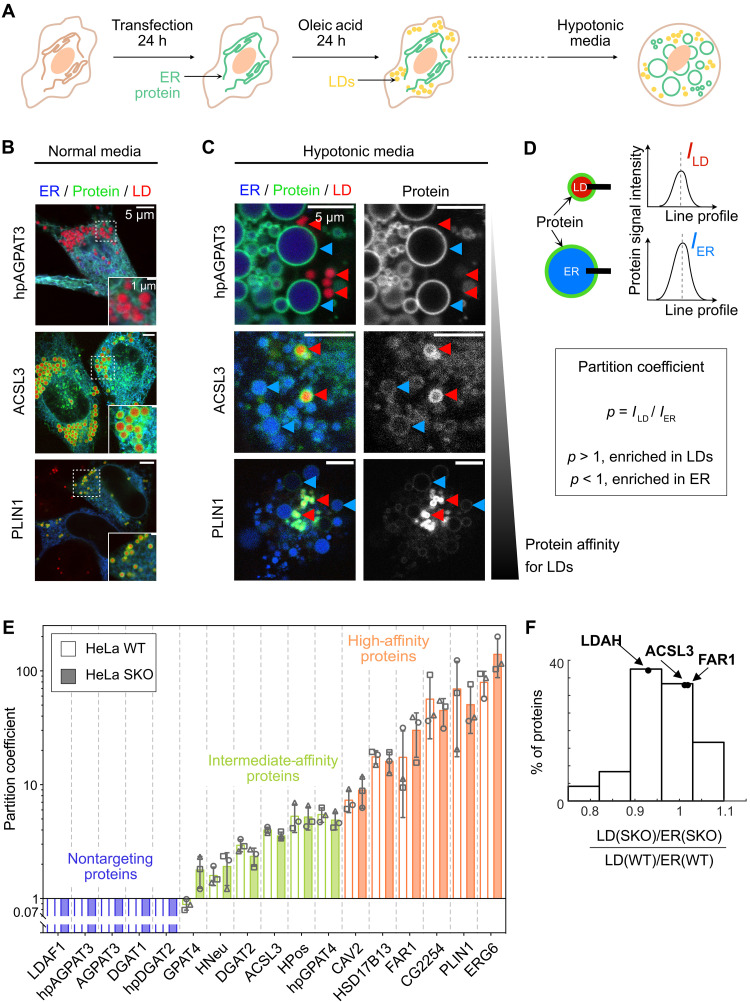
Class I proteins target LDs with a wide range of concentrations. (**A**) Schematic representation of the experimental protocol. HeLa cells are transfected with the protein of interest and an ER lumen marker for 24 hours. Cells are then exposed to OA (200 μM) for 24 hours and observed in the presence of an LD fluorescent reporter. h, hours. (**B** and **C**) Confocal microscopy images of cells transfected with hpAGPAT3, ACSL3, and PLIN1, from top to bottom. Channels: ER marker in blue (ERox), protein in green, and LD marker in red. Cells are observed in regular (B) or hypotonic media (C). In (C), red (resp. blue) arrows point to LDs (resp. ER) surface. (**D**) Quantification of the protein partition coefficient. Using a Gaussian fit, a line profile at the ER and LD equators gives respective protein intensities at the ER and LD surface. The partition coefficient is defined as the ratio between *I*_LD_ and *I*_ER_. (**E**) Partition coefficients for our 17 proteins subset are represented by the mean value as a bar and SD as gray whiskers, measured in HeLa WT (white bars) and HeLa SKO (colored bars). The average values of three independent experiments are represented by circle, square, and triangle gray symbols (between three and five cells for each experiment). Differences between WT and SKO partition coefficients for each protein are nonsignificant (unpaired *t* tests; see Table 1 for values). (**F**) Distribution of the relative abundance of class I proteins in LD fraction versus ER-enriched fraction between HeLa WT and HeLa SKO by proteomics (27 known class I proteins were identified).

HeLa cells were transfected with constructs of the target proteins fused to a fluorescent marker alongside a fluorescent marker for the ER lumen, ERox. Oleic acid (OA) was then fed to the cells for another 24 hours to induce large, mature LDs. This strategy focused only on the mature LD proteome and not on its early biogenesis stages (Fig. 1A). We visualized the proteins through fluorescence microscopy. Figure 1B shows three examples: (i) The hairpin of AGPAT3 is not found at mature LDs; (ii) ACSL3 shows high intensity on both the surface of LDs and the ER; and (iii) PLIN1 shows high intensity at the LDs and virtually no signal on the ER (fig. S1A).

Because of the ER network structure, it is not readily possible to accurately separate the ER and LD signals. Thus, we adopted an organelle swelling approach to improve the spatial separation of different organelles, which allowed us to better quantify ER versus LD surface signals. The osmotically induced swelling converts most bilayer-encircled organelles into vesicles, notably transforming the ER into giant ER vesicles (GERVs) (*45*) (fig. S1B). This strategy allowed reliable and quantitative separation of the ER and LD surfaces (Fig. 1C). This reduces the surface-to-volume ratio of the organelles, likely increasing the protein concentration in the swollen ER compared to the intact ER. Nonetheless, this strategy facilitates acute measurement and study of how the protein concentration in a relevant ER membrane affects protein recruitment to LDs. We determined the organelle partitioning coefficient *p* for each protein by calculating the ratio of intensity between the signal on the LD and the ER (Fig. 1D). When we plotted *p* for our 17 markers, we observed a gradual increase in the average partition coefficient across the group (Fig. 1E and figs. S1C and S2).

Partition coefficients varied over several orders of magnitude, revealing three different classes: nontargeting proteins, for which no signal was found on LDs (Fig. 1E, purple bar), reaching below our detection limit. Members in this class are LDAF1, AGPAT3, hpAGPAT3, DGAT1, and hpDGAT2. Intermediate-affinity proteins, such as GPAT4, HNeu, DGAT2, ACSL3, HPos, and hpGPAT4, were characterized by *p* < 5, and high-affinity proteins with *p* > 5 showing little to no signal on the ER were represented by CAV2, HSD17B13, FAR1, CG2254, PLIN1, and ERG6. In conclusion, this method allowed the acute assessment of the affinity of monotopic LD proteins and identified different classes within the tested ERTOLD proteins.

### Removal of seipin has a minor impact on the proteome of mature LDs

Positioned at the ER-LD junction, seipin can regulate protein trafficking between these organelles, especially during the early stages of LD formation and growth (*28*, *35*, *38*, *46*). Using our quantification method, we investigated the effect of seipin on the proteome of mature LDs by measuring the partition coefficients of the 17 selected proteins/peptides in a seipin knockout (SKO) HeLa cell line after 24-hour OA loading. We observed no significant changes in protein affinities between wild-type (WT) and SKO cells (Fig. 1E, fig. S2, and Table 1), suggesting that seipin removal does not have a substantial influence on the global proteome of mature LDs at 24 hours post-OA loading. This finding agrees with the recent finding of a late ERTOLD pathway independent of seipin (*28*).

**Table 1.** *P* values from the unpaired *t* test on the cell experiments between WT and SKO measurements. Unpaired *t* tests were performed on the average values of three independent measurements (Fig. 1E).

Protein	*P* value	Summary
GPAT4	0.0514	n.s.
HNeu	0.4617	n.s.
DGAT2	0.1424	n.s.
ACSL3	0.1059	n.s.
HPos	0.9466	n.s.
hpGPAT4	0.4425	n.s.
CAV2	0.4209	n.s.
HSD17B13	0.5449	n.s.
FAR1	0.2820	n.s.
CG2254	0.5792	n.s.
PLIN1	0.5922	n.s.
ERG6	0.1354	n.s.

Our assessment involved only a limited number of proteins for mature LDs and therefore aimed to expand our analysis to the mature LD proteome. We used mass spectrometry (MS) to assess global changes in the LD proteome between WT and SKO cells. Cells were fed with OA for an extended period, after which LDs were isolated and their proteome analyzed. We quantified the abundance of monotopic ER proteins in LD fractions and normalized them to their values in ER fractions, calculating a partitioning coefficient. The distribution of the partitioning ratio between SKO and WT cells for numerous established class I LD proteins showed a Gaussian-like distribution centered around 1, with a slight deenrichment of ~20% and an enrichment of 10% (Fig. 1F). Within our detection limit, we did not identify any protein that was excluded from or newly recruited to mature LDs upon seipin removal. These data also support that the proteome of mature LD fractions is only marginally affected by the absence of seipin (*47*) (fig. S3).

Some example proteins behave differently under WT versus SKO conditions. For example, AGPAT3 as well as its hairpin alone, did not visibly target LDs in WT or seipin-depleted cells. DGAT2 targeted LDs equally in WT and SKO cells, although its hairpin alone did not target LDs under either condition. In contrast, the hairpin of GPAT4 displayed a higher affinity for LDs than the full-length protein in both WT and SKO cells, raising the possibility that there are other targeting restriction domains in the full-length protein. Although most cells contained a uniform LD population, the LDs of GPAT4-transfected cells separated into two distinct populations, both in WT and SKO cells. Most LDs exhibited low levels of GPAT4 on the LD, whereas a subset displayed high intensity (fig. S4). Similar observations were reported in early studies in *Drosophila* S2 cells (*37*, *38*). On average, GPAT4 showed higher relocation to LDs in SKO cells than WT cells (Fig. 1E and fig. S4), similar to *Drosophila* cells (*37*, *38*).

Overall, our findings indicate that seipin does not act as a critical long-term regulator of the LD proteome. Only a small subset of proteins showed slight enrichment or displacement over prolonged periods without seipin. This suggests that other factors may be more determinant in regulating the ER-to-mature LD protein relocation.

### Intrinsic ER-to-LD protein partitioning estimation using droplet-embedded GERVs

During the 24-hour OA loading, one possibility is that the LD proteome undergoes dynamic remodeling due to factors such as protein degradation, recruitment by additional lipids and proteins, or structural changes of the ER-LD interface. As a result, the partitioning coefficient we measured may not capture the intrinsic propensity of a protein to partition between the ER and LDs. Furthermore, although kept to a maximum of 15 min, the osmotic swelling procedure might limit the validity of our observations. To address these potential caveats, we developed an experimental system called the droplet-embedded GERV (DEGERV). This is inspired by previously established techniques like droplet-embedded vesicles (DEVs) (*6*, *48*) and GERVs (*45*).

DEGERVs integrate artificial lipid droplets (aLDs) within native GERVs. This approach bypasses the metabolic complexities inherent to cellular systems, allowing for the direct visualization and quantification of ERTOLD proteins, independent of factors like seipin or the structural maturation of LDs. The technique provides “empty” LD surfaces, creating a simplified environment that can reveal the intrinsic propensity of any ER protein to relocate to LDs. Furthermore, DEGERVs offer insight into the determinants that guide ER proteins to LDs, free from the influence of metabolic processes that complicate the identification of LD protein targeting mechanisms. Although DEGERVs may not fully replicate the structural characteristics of ER-LD junctions in intact cells, they offer a robust platform for quantitatively assessing protein partitioning behavior. Specifically, they enable determination of the partition coefficient—i.e., the change in free energy (∆*G*) between the initial bilayer state and the final LD surface state—without considering energy barriers from the contact site. These barriers, which affect protein transfer efficiency or rate, likely vary on the basis of membrane curvature, seipin, protein interactions, and the cell’s state.

**Fig. 2.**
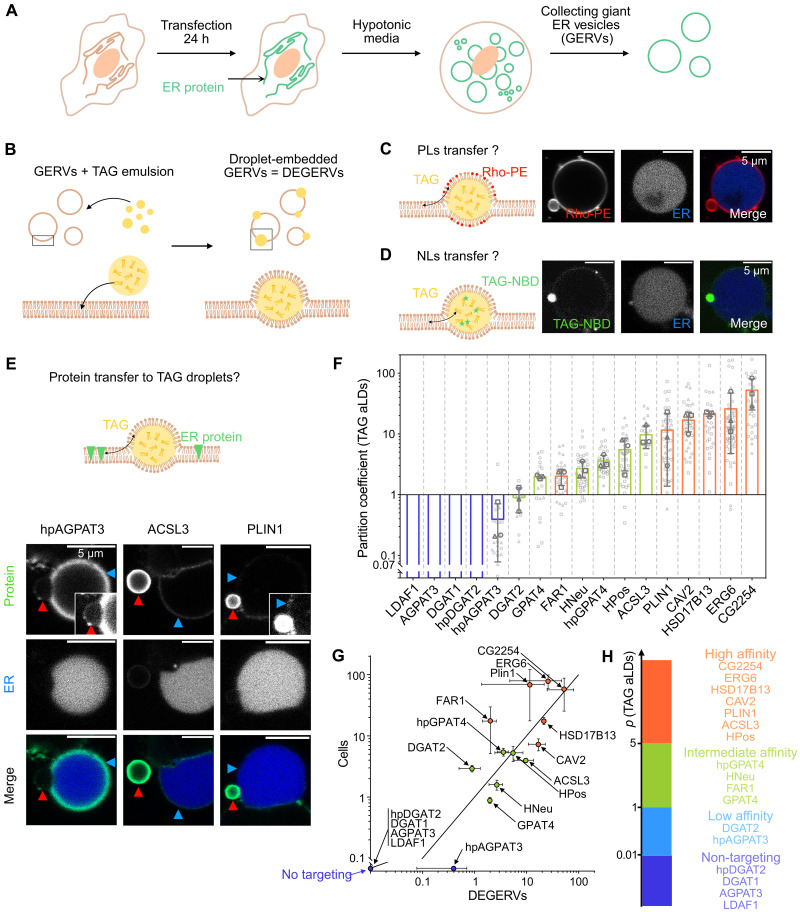
DEGERVs to dissect the regulatory mechanisms of LD targeting. (**A** and **B**) Schematic representation of the experimental protocol. (A) HeLa WT cells are transfected with the protein of interest and the ERox ER lumen marker for 24 hours. Cells are then exposed to hypotonic media, and GERVs are collected. (B) GERVs are mixed with a TAG-in-media droplets solution and spontaneously generate DEGERVs. Cartoon not to scale. (**C** and **D**) Characterization of TAG droplets incorporation in GERVs. (C) TAG-in-media emulsion was complemented with Rhodamine-DPPE [at 1:7000 (w/w) Rho-DPPE:TAG] to observe transfer of phospholipids (PLs) between the droplet and the GERV. (D) TAG-NBD-in-media emulsion was used, and a ring of the NBD signal was observed at the surface of the GERV, indicating a transfer of neutral lipids (NLs). (**E**) Images of DEGERVs prepared with cells transfected with hpAGPAT3, ACSL3, and PLIN1 (left to right). A low signal of hpAGPAT3 is observed at the TAG droplet (inset shows a higher brightness image of hpAGPAT3 on the LD). We observed a higher transfer of proteins for ACSL3 and PLIN1 (inset shows a higher brightness image of PLIN1 intensity on the ER). Red arrows point to LD, and blue arrows point to the ER surface. (**F**) The partition coefficient was measured as explained in Fig. 1D. It is represented with the mean value as a bar and SD as gray whiskers. The average values of three independent experiments are displayed as circle, square, and triangle gray symbols. All data points are represented in light gray (between 3 and 24 droplets for each experiment). (**G**) Average partition coefficient measured in cells (Fig. 1E) versus in DEGERVs; SDs are represented as whiskers. The plain line has a slope of 1. (**H**) Classification of proteins’ affinity for TAG droplets in DEGERVs.

To prepare DEGERVs, HeLa WT cells were placed in a hypotonic medium for 15 min and lysed (*45*). The resulting GERVs were visualized by the fluorescent ERox marker (Fig. 2A) and mixed with an aLD emulsion of TAG. These aLDs spontaneously fused with the GERVs (*48*, *49*), forming DEGERVs (Fig. 2B). The aLDs within GERVs typically measured between 1 and 3 μm, making them smaller than GERVs, with an average diameter of 9 ± 2 μm (fig. S5A).

To assess the efficient incorporation of aLDs into the GERVs, the aLDs were tagged with trace amounts of Rhodamine-DPPE [1,2-dipalmitoyl-sn-glycero-3-phosphoethanolamine; 1:7000 (w/w) to TAG]. Phospholipids from the aLDs readily transferred to the GERV membrane (Fig. 2C). Fluorescence recovery after photobleaching (FRAP) experiments indicated that these phospholipids diffuse between the GERV membrane and the aLD surface, ensuring the necessary fluidity and exchange of lipids between the two compartments (fig. S5B). Furthermore, when the aLDs were prepared with trace amounts of TAG-NBD (7-nitrobenz-2-oxa-1,3-diazol-4-yl), the fluorescent NBD signal also relocated to the GERV membrane, showing that TAG molecules were equilibrated between the aLDs and the GERV (Fig. 2D). Additional FRAP experiments revealed continuous TAG exchange between the GERV membrane and the aLDs (fig. S5C).

Next, we used this system to prepare GERVs from HeLa WT, which expressed our fluorescent marker proteins. The fluorescent protein concentration varied broadly due to differences in transfections, allowing for work with different protein concentrations in the ER. DEGERVs were then formed using the same TAG-based aLDs, and protein partitioning was assessed. We observed that proteins dynamically equilibrated between the GERV membrane and aLDs, as evidenced by the fluorescence recovery of the entire aLD following photobleaching (fig. S5D). The protein was perfectly diffusive on the ER membrane, and its partial fluorescence recovery at droplets was due to the limited ER reservoir capacity and its affinity for the TAG aLD (fig. S5D).

Most high-affinity and intermediate-affinity proteins, such as PLIN1 and ACSL3, spontaneously relocated to the aLDs (Fig. 2E). Proteins that do not specifically target LDs were generally absent from the surface of TAG-containing LDs. However, hpAGPAT3 consistently displayed a low-intensity signal on the surface of aLDs, although this protein did not typically visibly associate with LDs in cellular environments (inset of the hpAGPAT3 image, Fig. 2E).

We determined the partition coefficients for the 17 selected proteins/peptides and plotted them according to increasing levels (Fig. 2F and fig. S6A). We display nontargeting proteins below our detection sensitivity. The affinity order and range were consistent between cells and DEGERV experiments, with *p* ranging between 0.02 and ~100 (figs. S6B and S7, A and B). This is best illustrated by the plot of the partition coefficient in cells versus DEGERVs (Fig. 2, G and H), which showcases a linear correlation with a slope of 1 as a guide; a linear regression gave a slope of 1.32.

There are a few notable discrepancies between protein behavior in DEGERVs and cells, revealing regulatory mechanisms that may not be functional anymore in the DEGERV system (Table 2 and fig. S6B). DGAT2 and FAR1, above the correlation threshold, exhibit a lower affinity for TAG aLDs in DEGERVs compared to their behavior in cells. This suggests that, for these proteins, a minimal TAG droplet system does not faithfully replicate the partitioning as observed in cells. Factors such as membrane curvature, lipid composition, or protein-protein interactions may differ in DEGERVs than in cells, impinging on the partitioning behavior. Some discrepancies are found for high-affinity proteins PLIN1 and ERG6 (fig. S7B). These proteins’ partitioning coefficient error bars are huge, reflecting experimental variability in cells. Such variability likely arises from the fact that their levels in the ER are low and variable, and they also target the LD surface very efficiently. Therefore, dividing the LD signal by the ER signal leads to a significant, variable coefficient. On DEGERVs, the variability was less pronounced.

**Table 2.** *P* and *F* values from an ordinary one-way ANOVA test on the DEGERVs and cells experiments between WT, SKO, and DEGERV measurements. One-way ANOVA corrected for multiple comparisons (using the Tukey test) was performed on the average values of three independent measurements (fig. S6B).

Protein	*F*	*P* value	Summary
hpAGPAT3	4.436	0.0657	n.s.
GPAT4	9.007	0.0156	*
HNeu	2.618	0.1523	n.s.
DGAT2	21.30	0.0019	**
ACSL3	6.649	0.0301	*
HPos	0.01702	0.9832	n.s.
hpGPAT4	3.919	0.0815	n.s.
CAV2	5.003	0.0527	n.s.
HSD17B13	3.496	0.0985	n.s.
FAR1	5.705	0.0409	*
CG2254	0.1673	0.8498	n.s.
PLIN1	2.383	0.1731	n.s.
ERG6	8.129	0.0196	*

Conversely, proteins below the line demonstrate a higher affinity for aLDs in DEGERVs than in cells. This behavior is more straightforward to interpret as these proteins may inherently target LDs but are somehow restricted or displaced from the LD surface in cellular environments. A particularly notable example is hpAGPAT3, which is absent from LDs in cells and can yet be detected, albeit with low levels of DEGERVs. This is likely because DEGERVs initially contain no protein on the aLD surface, possibly converting some monotopic ER membrane proteins into weak ERTOLD cargoes. Once the LD surface becomes occupied, protein exclusion and competition may limit protein movement from the ER to LDs.

### Competition acts as a key regulator for protein partitioning in DEGERVs

**Fig. 3.**
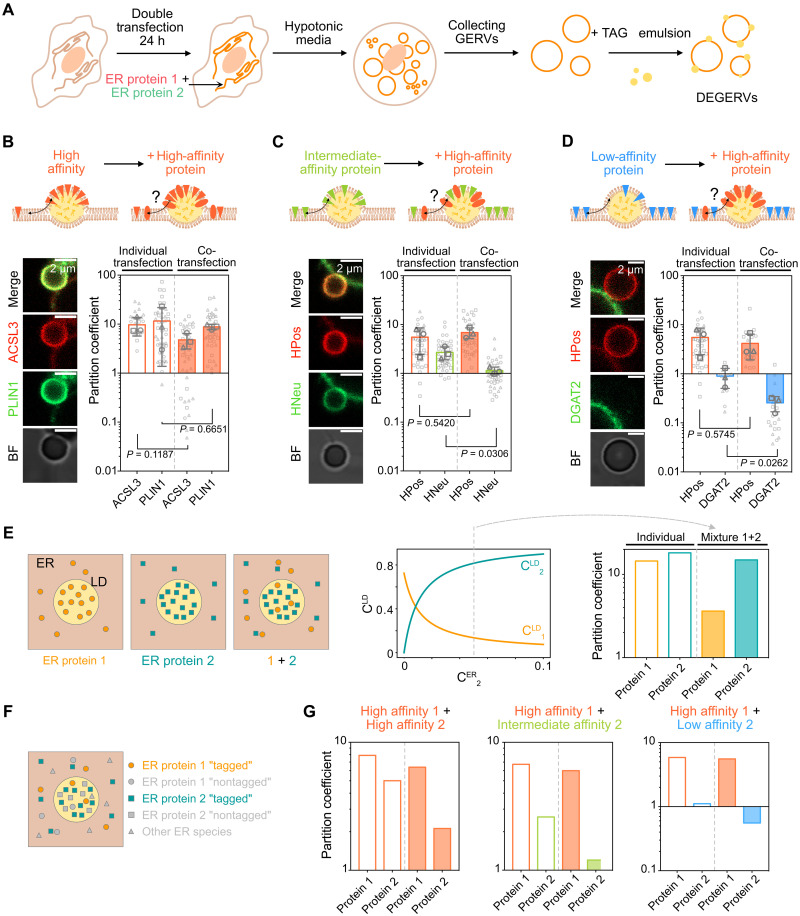
Protein competition affects the LD proteome. (**A**) Schematic representation of the experimental protocol, HeLa cells are cotransfected with two proteins. (**B** to **D**) We measured the partition coefficient of pairs of proteins with different TAG aLD affinities: high versus high-affinity couple ACSL3/PLIN1 (B), high versus intermediate HPos/HNeu (C), and high versus low HPos/DGAT2 (D). Cartoon not to scale. We show confocal images of an embedded droplet (left), and we compare the partition coefficient of both proteins when transfected individually (left; white bars, Fig. 2F) and upon cotransfection (right; colored bars). The average values of three independent experiments are represented as circle, square, and triangle gray symbols. All data points are presented in light gray (between 3 and 22 droplets for each experiment). Results of the unpaired *t* test are shown on the graph (see Tables 3 and 4). (**E**) Theoretical model describing steric protein-protein exclusion. For two proteins competing, the protein with the highest affinity will largely dominate LD relocation. The protein concentration on LDs $C_{i}^{\mathrm{LD}}$ is plotted as a function of $C_{2}^{\mathrm{ER}}$ according to Eq. 1 with parameters: $K_{1}=50$ $K_{2}=300$ , and $C_{1}^{\mathrm{ER}}=0.05$ . Partition coefficients plotted in the right correspond to $C_{2}^{\mathrm{ER}}=0.05$ . (**F**) We considered a pool of all endogenous proteins present in the ER, including proteins 1 and 2 (“nontagged”) and all other ER species, which will count as protein 3 in Eq. 1. Transfection refers to introducing a “tagged” population of proteins 1 and 2. (**G**) Predicted partition coefficient, for different affinity conditions, for proteins expressed individually (white bars) or coexpressed (colored bars). Under all conditions $K_{3}=10$ , overexpression of 1 and 2 was mimicked by taking $C_{i}^{\mathrm{ER}}$ times 10. For high/high affinity, $K_{1}=300$ and $K_{2}=100$ (left); for high/intermediate affinity, $K_{1}=150$ and $K_{2}=30$ (middle); and for high/low affinity, $K_{1}=100$ and $K_{2}=10$ (right).

To investigate the effect of competition between ER proteins for their relocation to LDs, we made DEGERVs from cells cotransfected with two ER proteins (Fig. 3A). We examined three distinct scenarios: (i) competition between two high-affinity proteins, ACSL3 and PLIN1, both of which exhibit high partitioning coefficients (*p* > 5 in DEGERVs; Fig. 3B); (ii) competition between a high-affinity peptide, HPos, and an intermediate-affinity peptide, HNeu (*p* > 5 and *p* < 5, respectively; Fig. 3C); and (iii) competition between a high-affinity peptide, HPos, and a low-affinity protein, DGAT2 (*p* < 1 in DEGERVs; Fig. 3D). In each case, we measured the partition coefficients for both proteins. We compared them to the respective *p* observed when expressed individually (Fig. 3, B to D, and Tables 3 and 4). High-affinity proteins share the aLD space; no significant change is observed in the *p* coefficients. However, we noted a subtle effect in the presence of PLIN1: The partitioning coefficients of ACSL3, HPos, and HSD17B13 decreased, slightly for the latter (fig. S8). This observation could indicate that high-affinity ER proteins with different affinities can compete to some extent for targeting LD and that PLIN1 predominates in LD relocation. On the other hand, when HPos was cotransfected with intermediate-affinity HNeu (Fig. 3C) or low-affinity DGAT2 (Fig. 3D), HPos efficiently displaced HNeu or DGAT2 from the aLDs. This result demonstrates that intermediate-affinity and low-affinity proteins are displaced from the LD surface in the DEGERV system by proteins with higher LD affinity.

**Table 3.** *P* values from the unpaired *t* test on the competition DEGERV experiments between individual transfection and cotransfection measurements. *t* tests were performed on the average values of three independent measurements (Fig. 3 and fig. S8).

Protein 1/Protein2	*P* value of protein 1	*P* value of protein 2	Summary 1/2
HPos/HNeu	0.5420	0.0306	n.s./*
HPos/PLIN1	0.4260	0.9611	n.s./n.s.
ACSL3/PLIN1	0.1187	0.6651	n.s./n.s.
HPos/DGAT2	0.5745	0.0262	n.s./*
PLIN1/HSD17B13	0.7468	0.1192	n.s./n.s.

**Table 4.** *P* values from the unpaired *t* test on the competition DEGERV experiments between protein 1 and 2 in individual transfection and in cotransfection measurements. *t* tests were performed on the average values of three independent measurements (Fig. 3 and fig. S8).

Protein 1/Protein2	*P* value for individual transf.	*P* value for cotransf.	Summary
HPos/HNeu	0.1955	0.0045	n.s./**
HPos/PLIN1	0.3792	0.1518	n.s./n.s.
ACSL3/PLIN1	0.7800	0.0250	n.s./*
HPos/DGAT2	0.0599	0.0395	n.s./*
PLIN1/HSD17B13	0.1728	0.4670	n.s./n.s.

Steric exclusion appeared to govern the exclusion of proteins from the LD surface, a process thermodynamically captured by the partitioning coefficient introduced in our system. To further evaluate whether our experimentally derived partitioning coefficient accounts for protein displacement, we developed a theoretical model to quantitatively assess its explanatory power.

We assume that proteins can freely diffuse between the LD and the ER, which we consider a protein reservoir. Each protein occupies a small area within the membrane, excluding other factors by steric repulsion. For simplicity, we assume that all proteins occupy the same space and do not interact with one another. At equilibrium, the normalized surface density of proteins i on the LD is (see the Supplementary Materials)

$$C_{i}^{\mathrm{LD}}=\frac{A_{i}}{1+\sum_{j}A_{j}}\;\text{with}\;A_{i}=K_{i}C_{i}^{\mathrm{ER}}$$ (1)

where $C_{i}^{\mathrm{ER}}$ is the concentration of a protein i in the ER and $K_{i}$ is the affinity constant of the LD binding reaction. The propensity of a protein to relocate to the LD surface depends on $A_{i}$ , the product of these two quantities. $K_{i}$ exponentially depends on the free energy difference of the protein between the ER and LD. If $K_{i}>1$ (resp. $0\leq K_{i}<1$ ), the protein preferentially relocates to the LD (resp. stays in the ER). The sum in the denominator of Eq. 1 accounts for the steric repulsions between proteins on LDs: It represents the combined influence of all proteins competing for space on the LD surface. The density of each protein type depends on the relative values of the parameters {$A_{j}$} . To illustrate the effect of the mutual steric exclusion between two proteins, we first represented a simplified situation in which only one or two ER proteins can move onto the LDs (Fig. 3E). The simulation showed that marginal differences in the LD affinities were sufficient to compete out the protein with lower affinity, even when both competitors had high LD affinity by themselves.

Next, we used this model to simulate our experiments and asked how the simultaneous incorporation of multiple proteins that relocate from the ER to LDs contributes to steric exclusion (Fig. 3F; see the Supplementary Materials). To simulate the expression levels of the proteins, we introduced a scaling factor reflecting the protein density or concentration in the ER. Figure 3G shows the theoretically predicted partition coefficient for different affinities of two proteins, expressed individually or together. Notably, the model successfully recapitulated our experimental findings using DEGERVs. In particular, proteins like DGAT2, which have a relatively lower affinity for TAG aLDs, were almost entirely excluded under certain conditions (Fig. 3, D and G). This result underscores that competition for LD surface area through steric exclusion, according to the proteins’ partition coefficient, strongly influences the composition of the LD proteome. ER proteins with lower affinity for TAG aLDs, such as AGPAT3 or DGAT2, may be entirely excluded from LDs by the sole action of steric exclusion.

### The ERTOLD pathway is critically regulated by protein exclusion mechanisms

**Fig. 4.**
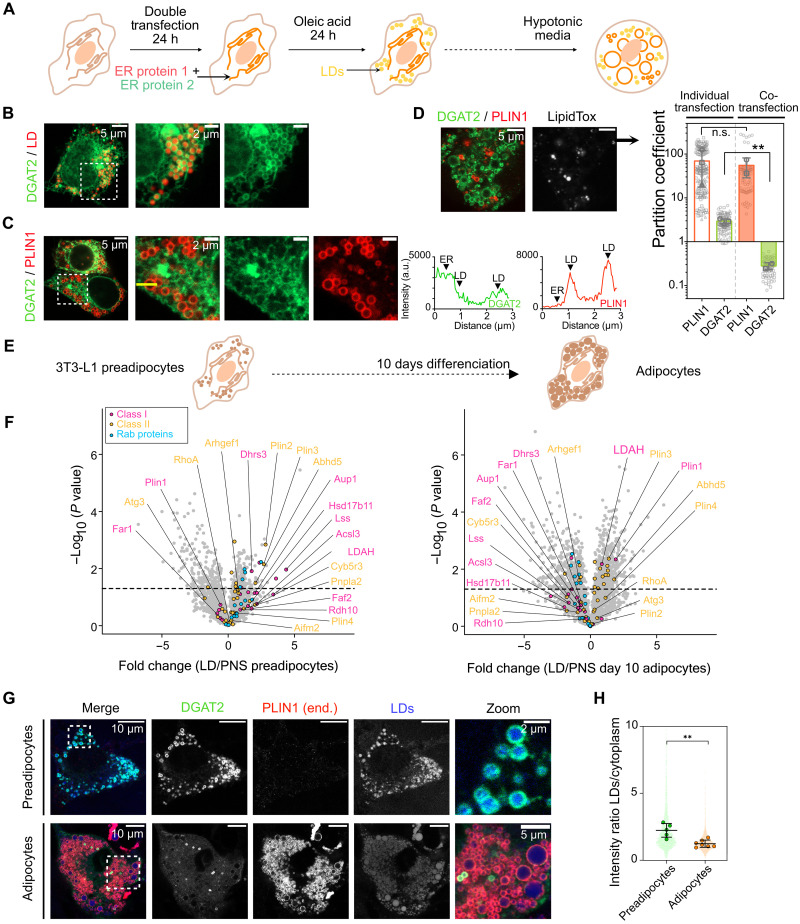
The ERTOLD pathway is highly sensitive to protein exclusion mechanisms. (**A**) Schematic representation of the experimental protocol: HeLa cells are cotransfected with two proteins. (**B**) Confocal microscopy images of HeLa WT cells transfected with DGAT2 only. (**C** and **D**) Confocal microscopy images of cells cotransfected with PLIN1 and DGAT2, in regular (C) and hypotonic (D) media, with line profiles in both channels along the yellow line (C). We compare the partition coefficients [(D), right] of individual transfected protein (white bars, Fig. 1E) and cotransfected (colored). Average values of three (resp. two for the cotransfection) independent experiments are represented as circle, square, and triangle gray symbols. All data points are presented in light gray (between 73 and 80 droplets total, on four to five cells for each experiment). Results of the unpaired *t* test are shown (see Table 5 for values). a.u., arbitrary units. (**E**) Schematic representation of the experimental protocol using 3T3-L1 cells. (**F**) Relative abundance of proteins in LD fraction versus PNS fraction analyzed by proteomics in preadipocytes (left) and 10 days adipocytes (right) as a volcano plot: statistical significance −log_10_(*P* value) as a function of the fold change of the relative LD abundance of class I (pink), class II (orange), and Rab family (blue) proteins. Proteins above the dashed line correspond to a *P* value of <0.05. (**G**) Confocal microscopy images of overexpressed DGAT2 (green) and endogenously expressed PLIN1 (red, by immunofluorescence) in preadipocytes, exposed to 200 μM OA overnight, and 10-day adipocytes. LDs are labeled with LipidToxDeepRed (blue). (**H**) Quantification of DGAT2 intensity at LDs, normalized by intensity in the ER, in preadipocytes and adipocytes. Average per cell, measured from two independent experiments, are large circles; individual LD values are shown in the background. The *y* axis limit of 10 prevents displaying 50 individual data points that range from 20.

We investigated the influence of steric competition on the LD proteome in living cells. We cotransfected HeLa WT cells for 24 hours with pairs of proteins exhibiting different affinities for LDs. Cells were subsequently treated with OA for 24 hours to induce mature LD formation (Fig. 4A). Upon coexpression, the two high-affinity proteins PLIN1 and HSD17B13 largely colocalized on LDs. However, smaller LDs predominantly displayed the PLIN1 signal, suggesting a temporal or spatial dominance of PLIN1 at specific stages of LD maturation (fig. S9, A and G).

When expressed alone, the intermediate-affinity protein DGAT2 localized to LDs (Fig. 4B). Coexpression with PLIN1 resulted in the near-complete displacement of DGAT2 from LDs back to the ER, consistent with our theoretical predictions and DEGERV experimental results (Fig. 4, C and D, and Table 5). In contrast, DGAT2 was not completely excluded by the artificial peptide HPos (fig. S9, B and G), which we classified as an intermediate-affinity protein with lower LD affinity than PLIN1. DGAT2 was, however, displaced by hpGPAT4, a protein with a higher partition coefficient (*p*) (fig. S9, C and G). As a control, DGAT2 remained on LDs when coexpressed with hpAGPAT3, a nontargeting protein in cells and weak targeting in GERVs (fig. S9D).

**Table 5.** *P* values from the unpaired *t* test on the competition experiments in cells between individual transfection and cotransfection measurements. *t* tests were performed on the average values of two or three independent measurements (Fig. 4 and fig. S9).

Protein 1/Protein2	*P* value of protein 1	*P* value of protein 2	Summary 1/2
DGAT2/HPos	0.6888	0.0151	n.s./*
DGAT2/PLIN1	0.0022	0.7516	**/n.s.

Furthermore, we observed that other intermediate-affinity proteins, such as hpGPAT4 and HNeu, were excluded from LDs when coexpressed with higher-affinity proteins HSD17B13 or PLIN1, respectively (fig. S9, E to G). These findings support a model in which steric competition, modulated by relative LD affinity, shapes the composition of the LD ERTOLD proteome.

Our analyses open the possibility that affinity-based competition between different ERTOLD cargoes may be used to tune the LD surface composition to meet cellular needs. Collectively, our theoretical and experimental results would predict that proteins with lower affinity for TAG-LDs, such as DGAT2, may, under certain circumstances, be dynamically excluded from LDs. Regulation of LD surface composition may, for instance, be achieved by controlling the expression levels of competing LD proteins. For example, PLIN1 is not expressed in preadipocytes, but its mRNA and protein levels increase rapidly when preadipocytes differentiate into adipocytes. Previous works have shown that PLIN1 efficiently excludes CYTOLD proteins such as PLIN2, PLIN3, and PLIN4 from LDs (*29*, *44*, *50*), which have lower LD affinity than PLIN1. We therefore used adipocytes to test the validity of our model predicting the exclusion of lower-affinity by high-affinity ERTOLD cargo. Specifically, we hypothesized that PLIN1 might displace lower-affinity ERTOLD cargo upon differentiation.

To compare the LD protein composition in the absence and presence of endogenously expressed PLIN1 in an unbiased manner by proteomics, we prepared LDs from 3T3-L1 preadipocytes after OA loading and from white adipocytes 10 days after differentiation (Fig. 4E). The volcano plots in Fig. 4F (fig. S10, A and B) and the box plots in fig. S10C show the significant enrichment of PLIN1 in LDs of adipocytes, compared to the levels in the postnuclear supernatant (PNS). Agreeing with our predictions, we found a global decrease in the recruitment of ERTOLD proteins in the LD-enriched fraction from differentiated cells (Fig. 4E and fig. S10C). These data suggest that the recruitment of high-affinity PLIN1 to TAG LDs competes with other monotopic proteins. It is important to note that competition does not necessarily mean a protein is wholly excluded from LDs. Instead, its abundance on LDs is limited, depending on its affinity for LDs. This controlled restriction of protein levels on LDs could be a mechanism to fine-tune the speed of specific biochemical reactions.

Next, we visualize overexpressed DGAT2-EGFP alongside endogenous PLIN1 in fixed 3T3-L1 preadipocyte and adipocyte cells. In preadipocytes, DGAT2 effectively moved to LDs induced by OA treatment (Fig. 4G). Adipocytes, with their smaller surface-to-volume ratio, favoring competition, showed virtually no DGAT2 on LDs after several days postdifferentiation (Fig. 4G). Quantification of LD-localized intensity relative to ER intensity (Fig. 4H) confirmed a significant decrease in adipocytes compared to preadipocytes. As anticipated, endogenous PLIN1 was absent in preadipocytes but highly abundant in adipocyte LDs wherein DGAT2 was excluded (Fig. 4G), likely preventing LD association with overexpressed DGAT2. Only OA treatment of mature adipocytes could redirect DGAT2 to some LDs (fig. S10D), suggesting that some lipogenesis factors may be required for DGAT2 LD targeting.

These findings further support a mechanism by which high-affinity proteins such as PLIN1 compete with lower-affinity ERTOLD proteins like DGAT2 by steric hindrance.

## DISCUSSION

Obtaining an accurate inventory of LD proteins is challenging due to the diversity in LD size, composition, and metabolic state (*11*). A dynamic view of the LD proteome is essential as LDs evolve from nucleation to maturation, performing various functions across different timescales and metabolic conditions. Protein targeting to LDs is not binary; proteins accumulate to varying concentrations on LD surfaces, influencing the rate of LD-related processes. Our study highlights the role of steric repulsion in controlling protein concentration and nonselective recruitment (*16*) to LDs. Protein affinity for LDs depends on the energy difference between the LD monolayer and the ER bilayer, with high-affinity proteins having priority in targeting LDs—unless regulated by an energy barrier, potentially mediated by seipin during the early stage of LDs. This energy balance shifts with the cell’s metabolic state and may vary by cell type.

Several thermodynamic and biophysical variables, including the concentration of the ER protein reservoir and the available surface area of LDs, influence the protein affinity for LDs measured in this study. These parameters were challenging to control directly; however, we leveraged the natural variability in transfection efficiency to modulate the protein concentration and the size distribution of aLDs incorporated into GERVs. We observed no clear correlation with the measured partition coefficients within the explored droplet size ranges and ER protein concentration (fig. S11). Variability was especially pronounced at higher affinities.

We classified the affinities of ER proteins targeting LDs under energy-rich conditions, with cells exposed to high concentrations of OA for 24 hours. This represents a prolonged, steady-state condition compared to the shorter LD growth period. Because even slight differences in affinity can lead to significant protein exclusion (Fig. 3), and the measured affinities vary by several orders of magnitude, cells must use regulatory mechanisms to maintain LD proteome plasticity, enabling them to adapt effectively to environmental fluctuations.

Protein levels in LDs must follow a precise timeline to ensure proper metabolic responses. Early (biogenesis) and late (mature LD) ERTOLD pathways have been identified in *Drosophila* cells (*28*), and similar mechanisms may exist in mammalian cells. In the early pathway, proteins move from the ER to LDs by passing through the seipin complex. In the late pathway, seipin is absent at the ER-LD connection, allowing proteins like GPAT4, which fail to target nascent LDs, to use this route (*28*, *38*). Our findings suggest that seipin has a marginal impact on the proteome of steady-state LDs, supporting the idea of a late pathway in mammals. We speculate that seipin mainly protects the LD proteome during early growth, ensuring lipid-metabolizing enzymes, such as DGAT2, can drive LD biogenesis and maturation without being displaced by high-affinity proteins. Although DGAT2 may initially target early LDs, it could later be displaced to the ER as LDs mature. High-affinity proteins, recruited later, may stabilize LDs by controlling the further entry of proteins. This aligns with our observations of DGAT2 localization in preadipocytes and adipocytes. In preadipocytes, DGAT2 promotes LD growth by converting DAG to TAG (*36*). However, DGAT2 is excluded from the larger PLIN1 LD subpopulation in adipocytes, where growth is driven by the PLIN1/CIDE-dependent fusion (*51*, *52*) of smaller LDs rather than DGAT2 activity.

The regulation of protein relocation to the LD surface is critical for controlling LD function. This regulation is likely a combination of genetic coding and metabolic control, where cells activate the translation of specific proteins under particular conditions or cues. The affinities of proteins for LDs may be inherently programmed into their genetic expression profiles, enabling cells to adjust LD-associated protein levels in response to various demands, such as those encountered during the cell cycle or under stress conditions like metabolic or chronic stress. However, protein affinity for LDs is not solely determined by transcriptional control; posttranslational modifications, such as phosphorylation or palmitoylation, play a crucial role in fine-tuning these interactions in response to environmental signals (*37*, *53*–*55*). For example, during nutrient deprivation, the phosphorylation of PLINs may reduce their affinity for LDs, facilitating the recruitment of lipolytic enzymes necessary for lipid mobilization (*55*, *56*). This genetic expression and posttranslational modification allow cells to regulate LD function dynamically.

Last, when comparing our results between cells and DEGERVs, we noticed some discrepancies that warrant further investigation. For proteins like DGAT2 and FAR1, a minimal TAG LD system did not fully replicate the LD relocation observed in cells. This suggests that our system may currently miss essential factors that would enable it to fully mimic the cell condition as additional factors may influence protein affinity for LDs. Potential contributors include altered LD lipid chemistry, the highly curved bilayer-LD neck acting as an energy barrier for diffusion, and other retention factors within the ER. The DEGERV platform provides an ideal system for fine-tuning these properties further and better exploring their impact on the ERTOLD pathway.

## MATERIALS AND METHODS

### Cell culture

HeLa WT and SKO cells (gifted by R. Yang) were maintained in high glucose (4.5 g/liter) with stabilized glutamine and with sodium pyruvate Dulbecco’s modified Eagle’s medium (DMEM) (Dutscher) supplemented with 10% fetal bovine serum (FBS) and 1% penicillin/streptomycin (PS) (GibcoBRL). Cells were cultivated 48 hours at 37°C with 5% CO_2_. To induce feeding conditions and LD formation, cells were incubated for 24 hours with DMEM supplemented with fatty acids conjugated to bovine serum albumin (BSA) (1%, v/v), used at a concentration of 200 μM OA. Cells were regularly tested, and no contamination for mycoplasma was detected.

### Cell transfections and plasmids

Cells were seeded in MatTek 3.5-mm coverslip bottom dishes (MatTek Corp. Ashland, MA) for 24 hours before transfections. Cells were transfected with 2 μg of the indicated plasmid using jetPEI transfection reagent (PolyPlus, #10110 N). Cells were transfected with different plasmids fused with fluorescent protein constructs 24 hours, before 24-hour feeding with OA for cells experiments (Figs. 1 and 4), or before giant organelles collection for DEGERV experiments (no feeding, Figs. 2 and 3). The following is a list of the used plasmids. ERoxBFP was a gift from E. Snapp (Addgene plasmid #68126; http://n2t.net/addgene:68126; RRID:Addgene 68126). EGFP-DGAT1, EGFP-DGAT2, and mCherry-AGPAT3 were gifts from R. Yang (University of New South Wales, Sydney, Australia). EGFP-FAR1 and ACSL3-mCherry were gifts from J. Füllekrug (Heidelberg University, Heidelberg, Germany). EGFP-hpAGPAT3 and EGFP-hpDGAT2 were designed and purchased from VectorBuilder (Germany). EGFP-PLIN1 and mCherry-PLIN1 were gifts from D. B. Savage (University of Cambridge, Cambridge, UK). ERG6-EGFP was a gift from M. Henne (UT Southwestern Medical Center, Dallas, TX, USA). HSD17B13-turboGFP was a gift from Y. Rotman (NIDDK, ML, USA). LDAF1-turboGFP was a gift from M. Bohnert (University of Münster, Münster, Germany). CAV2-EGFP, EGFP-HNeu, and mOrange-HPos were gifts from A. Pol (Universitat de Barcelona, Barcelona, Spain). EYFP-CG2254 was a gift from M. Beller (Heinrich Heine University, Düsseldorf, Germany). mApple-hpGPAT4 was a gift from S. Cohen (University of North Carolina at Chapel Hill, Chapel Hill, NC, USA). EGFP-GPAT4 was a gift from S. Kersten (Cornell University, Ithaca, NY, USA).

### GERV production and extraction

After transfection (and OA feeding for cell experiments), the cultured cells were transferred into a hypotonic culture media DMEM:H_2_O (5:95%, v/v) at pH 7.4, at 37°C, 5% CO_2_, for 15 min, to induce cell volume increase and giant organelle vesicles (GOVs). For cell experiments (Figs. 1 and 4), observation was performed at this step directly in the MatTek coverslip bottom dishes. For DEGERV experiments (Figs. 2 and 3), cells were mechanically lysed by extensive pipetting, and the solution was filtered at 10 μm to extract GOVs, including GERVs.

### DEGERV production

Droplets were made using an oil-in-hypotonic media emulsion: 8 μl of triolein (or TAG in the paper; G7793, Sigma-Aldrich) was mixed with 300 μl of hypotonic media. The solution is subsequently sonicated and vortexed to form small droplets. The emulsion is mixed with the GERV solution by gentle pipetting and incubated on the bench for 10 min to form DEGERVs (Fig. 2B). For the phospholipid transfer experiment (Fig. 2C), a dried film of Rhodamine-DPPE was mixed in triolein at 1:7000 (w/w) Rho-DPPE:TAG, before DEGERV preparation. For the neutral lipid transfer experiment (Fig. 2D), a solution of TAG-NBD:TAG 1:50 (v/v) was used to prepare the emulsion and DEGERVs (TAG-NBD: 6285, Setareh Biotech). With all approaches, the DEGERV sample was then placed on a glass coverslip pretreated with 10% (w/w) BSA and washed five times with hypotonic media, and it was then observed by confocal fluorescence microscopy.

### Fluorescent probes

For cells experiments, LDs were tagged with HCS LipidTox Deep Red Neutral Lipid Stain (0.05%, v/v; catalog no. H34477, Thermo Fisher Scientific) for EGFP and EYFP tagged proteins or Bodipy 493/503 (0.05%, v/v; Thermo Fisher Scientific) for mOrange, mApple, and mCherry tagged proteins.

### Confocal microscopy imaging

All micrographs were captured using a Carl Zeiss LSM800 microscope with an oil-immersed ×63 objective. EGFP, EYFP, NBD, and Bodipy 493/503 fluorescence was excited at 488 nm, and emission was detected between 510 and 550 nm. In contrast, mOrange, mApple, and mCherry tagged protein fluorescence was excited at 561 nm, and emission was detected between 580 and 650 nm. Deep red fluorescence was excited at 640 nm and was detected above 650 nm. BFP fluorescence was excited at 405 nm and detected below 500 nm. For both cell and DEGERV experiments, *Z*-stacks were acquired with a stack gap of 0.5 to 0.8 μm to cover the entire object of interest. All fluorescence signals were analyzed with ImageJ (see the Data analysis section below).

### FRAP experiment in DEGERVs

FRAP experiments were performed by bleaching either the total surface of a droplet or a part of the ER bilayer (fig. S5). Fluorescent proteins or lipids were bleached. Then, the recovery of signals was monitored. The FRAP curves were normalized by the fluorescence before bleaching and immediately after bleaching in the region of interest.

### Data analysis

All data acquired were analyzed with ImageJ. A 5-pixel-thick line is plotted at the ER and LD equator lines; the line profiles obtained at these lines are fitted by a Gaussian, whose maximum gives respectively protein intensities at the ER and LD surface. For cell experiments (Figs. 1 and 4), intensities on at least eight ER-vesicles are averaged to measure *I*_ER_ of one cell; and individual LD intensities *I*_LD_ are measured (at least 10 LDs per cell). For DEGERV experiments (Figs. 2 and 3), *I*_ER_ was averaged from measurements on five images close to the GERV equator, and individual LD intensities *I*_LD_ were measured. Partition coefficient is defined as the ratio between *I*_LD_ and *I*_ER_ and can be measured for individual droplets. For proteins that did not target LDs at all, we estimated that it is hard to measure any signal intensity at the droplet surface, which is lower than 1.5 times the background noise. This arbitrary limit for LD intensity, when divided by the ER intensity, gave us values between 0.01 and 0.02. Therefore, we consider our detection limit to be around 0.02. Background intensity (measured on a 100 × 100–pixel extracellular region) was subtracted from *I*_ER_ and *I*_LD_.

### Statistical analysis

Statistical comparisons were made on the mean values of the three independent experiments, using a nonparametric *t* test or a one-way analysis of variance (ANOVA) [GraphPad Prism; unless the *P* value is directly written on graph, ** indicates *P* < 0.001, * indicates *P* < 0.05, and not significant (n.s.) indicates *P* > 0.05].

### Data representation

GraphPad Prism was used to represent most graphs. Most of them are represented on a log scale with the mean value as a bar and the SD as gray whiskers. Average values of three independent experiments are described as circle, square, and triangle gray symbols; and all individual data points are represented in light gray symbols (respectively circles, squares, and triangles).

### Theoretical model

See the Supplementary Text.

### Preadipocyte and adipocyte cell culture and transfection

3T3-L1 preadipocytes were obtained from the American Type Culture Collection (CL-173) and cultured at subconfluency in high glucose DMEM containing stabilized glutamine and sodium pyruvate, supplemented with 10% FBS (Sigma-Aldrich, N4637) and 1% PS (Sigma-Aldrich, #P4333), at 37°C and 5% CO_2_. For differentiation into adipocytes, the cells were seeded to grow to confluence the following day and maintained at confluence for 48 hours. Differentiation was induced by adding an adipocyte differentiation medium: DMEM supplemented with 10% FBS (Sigma-Aldrich, #F7524), 1% PS, 172 nM (1:10,000) bovine insulin (Sigma-Aldrich, #I0516), 500 μM 3-isobutyl-1-methylxanthine (Sigma-Aldrich, #I5879), and 0.25 mM dexamethasone (Gbiosciences, #API-04). After 48 hours, the differentiation medium was replaced with DMEM, 10% FBS, 1% PS, and 172 nM bovine insulin, which was then replaced after 2 days with DMEM and 10% FBS, refreshed every 2 days thereafter. The cells were used for experiments on day 10 after differentiation began. Preadipocytes and adipocytes were transfected with EGFP-DGAT2 via electroporation and then incubated overnight in the same medium, containing the 200 μM OA/BSA complex (described before) to induce LD formation when indicated.

### Preadipocyte and adipocyte staining for immunofluorescence, imaging, and analysis

Transfected preadipocytes and differentiated adipocytes were fixed with 4% paraformaldehyde in Dulbecco’s Phosphate-Buffered Saline (DPBS) for 20 min and then permeabilized with a permeabilizing buffer (DPBS containing saponin; catalog no. 10294440, Fisher Scientific) at a concentration of 0.025% w/v and gelatin from cold water fish skin (catalog no. G7041, Sigma-Aldrich) at a concentration of 0.7% w/v for 20 min at 37°C. Cells were subsequently incubated with goat anti-PLIN1 antibody (Abcam, #ab60269) for 2 hours, followed by washing three times with permeabilizing buffer for 5 min. Cells were then incubated with the secondary (Donkey anti-Goat Alexa Fluor 546, Invitrogen, #A-11056) and LipidToxDeepRed neutral lipid stain for 90 min. Images were acquired on the confocal microscope previously described.

Images were first processed on Fiji (ImageJ) to split channels and remove the noise from the LD channel. LDs were segmented on Python using a custom Stardist model (*57*) and cells using the “cyto2” model from CellPose 2.0 (*58*). Images and masks were processed on CellProfiler 3.0 (*59*). To analyze DGAT2 signal intensity, we measured the signal intensity using a 2-pixel-wide ring around LDs, divided by the signal intensity from the rest of the cell, excluding LDs and 4 pixels around these objects. Data from CellProfiler were analyzed using Python 3.10 and plotted in GraphPad Prism.

### LD floatation

For proteomic analysis, we isolated LDs from 3T3-L1 preadipocytes (three 10-cm plates combined per sample) loaded with 500 μM sodium oleate for 8 hours and from 3T3-L1 adipocytes (one 10-cm plate per sample) after 10 days of differentiation. LD floatation was done as in Majchrzak *et al.* (*29*) by sucrose density centrifugation. Briefly, the cells were washed with and scraped into ice-cold phosphate-buffered saline (PBS), pelleted at 600*g*, 4°C for 5 min, and resuspended in 1 ml of PBS supplemented with protease inhibitors (cOmplete, Mini, EDTA-free Protease Inhibitor Cocktail; Merck) and phosphatase inhibitors (Pierce, Thermo Fisher Scientific, A32955). The cells were lysed by 20 passages through a bead homogenizer (16-μm bead, Isobiotec), and the lysate was clarified at 600*g*, 4°C for 10 min. The PNS was mixed with 60% sucrose/PBS (w/v) to a final concentration of 12% sucrose, transferred to an ultracentrifuge tube (5 mm by 41 mm, Beckman-Coulter, #344090) and centrifuged in an MLS-50 rotor (Beckman-Coulter) at 100,000*g*, 4°C for 1 hour. The tubes were sealed and flash frozen in liquid nitrogen. The top fraction containing LDs and LD-associated cofloating membranes was collected by cutting the frozen tube right at the level below visible LDs. The tube was thawed to collect the middle soluble fraction (cytosol), and the remaining pellet containing intracellular membranes was washed once with PBS and then resuspended in PBS. The protein concentration was determined by BCA for each fraction. Equal amounts of proteins in the LD-enriched fraction, and the corresponding PNS (input) from three independent samples were processed.

### Proteomics

The samples were trypsin digested and purified for proteomics by the PreOmics kit from iST, following the manufacturer’s instructions. Five micrograms of protein from the LD and the corresponding postnuclear fraction was used as the input. The samples were processed as triplicates from three independent experiments.

LD and ER fractions from WT or SKO cells were normalized according to protein concentration (Fig. 1F and fig. S3). Peptides were generated following the protocol of the iST sample Preparation Kit (preOmics). Peptides were transferred to a glass vial, and 10 μl was used for reversed-phase chromatography on a Thermo Ultimate 3000 RSLCnano system, connected to a Q Exactive PLUS mass spectrometer (Thermo Fisher Scientific) via a nano-electrospray ion source. Separation was achieved using a 50-cm PepMap C18 Easy-Spray column (Thermo Fisher Scientific) with a 75-μm inner diameter, maintained at 40°C. Peptides were eluted with a linear gradient of acetonitrile (12 to 35% in 0.1% formic acid) over 80 min at a constant flow rate of 250 nl/min, followed by an increase to 60% over 20 min, and lastly to 90% over 10 min. Eluted peptides were directly electrosprayed into the mass spectrometer. Mass spectra were acquired on the Q Exactive PLUS in data-dependent mode, alternating between full-scan MS and up to 10 data-dependent MS/MS scans. The maximum injection time for full scans was 50 ms, with a target value of 3,000,000 at a resolution of 70,000 at mass/charge ratio (*m/z*) 200. The 10 most intense multiply charged ions (*z* ≥ 2) from the survey scan were selected with an isolation width of 1.6 Th and fragmented using higher-energy collision dissociation (*60*) with normalized collision energies of 27. Target values for MS/MS were set to 100,000, with a maximum injection time of 80 ms at a resolution of 17,500 at *m/z* 200. To avoid repetitive sequencing, dynamic exclusion was set to 20 s. The resulting MS and MS/MS spectra were analyzed using MaxQuant (version V2.4.14.0, www.maxquant.org) (*61*, *62*) and Perseus (V2.0.11.0, www.maxquant.org/perseus). Plots were performed with the R software package (www.r-project.org/; RRID: SCR_001905). All samples were processed as triplicates from three independent experiments. The MS proteomics data have been deposited in the ProteomeXchange Consortium via the PRIDE partner repository with the dataset identifier PXD064671 (*63*).

Equal amounts (2.5 μg) of protein from the PNS and fractions enriched for LDs from day 10 adipocytes and sodium oleate–treated preadipocytes (three samples per group) were processed using the iST Sample Preparation Kit (PreOmics, P.O.00001), according to the manufacturer’s manual. Briefly, samples were digested by trypsin/LysC, digests were purified on the kit columns and dried under vacuum (SpeedVac). The resulting peptides were resuspended and analyzed by LC-MS/MS (liquid chromatography–tandem mass spectrometry) using reversed-phase chromatography on a Thermo UltiMate 3000 SLCnano system and sprayed into a TimsTOF HT mass spectrometer (Bruker Corporation, Bremen). For the chromatography, an Aurora Gen3 C18 column (25 cm by 75 μm by 1.6 μm) with CSI emitter (Ionoptics, Australia) at 40°C was used. Peptides were eluted from the column via a linear acetonitrile gradient from 10 to 35% in 0.1% formic acid for 44 min at a constant flow rate of 300 nl/min. Afterward, the gradient was increased to 50% buffer B for 7 min, followed by a 4-min increase to 85% buffer B. Peptides were sprayed into the TimsTOF HT mass spectrometer through a Captive Spray Ion source at an electrospray voltage of 1.6 kV and a DryGas (3 liters/min). The mass spectrometer was operated in positive ion mode and a MS range from 100 to 1700 *m*/*z* using the Parallel Accumulation–Serial Fragmentation (PASEF) scan mode. Ion mobility was ramped from 0.7 Vs/cm^2^ to 1.5 in 100 ms with an accumulation time set of 100 ms. Ten PASEF ramps per cycle resulted in a duty cycle time of 1.17 s with a dynamic exclusion time of 0.4 min. The precursor ion charge state was limited from 0 to 5. The resulting MS and MS/MS spectra were analyzed using MaxQuant [V2.4.11.0, www.maxquant.org/ (*61*, *62*)] and Perseus (V2.0.11.0, www.maxquant.org/perseus). For the label-free quantification, the LD-enriched samples and the PNS samples were compared (*64*). This analysis yields an enrichment factor of the LD sample as described previously for yeast vacuoles (*65*). Plots were performed with the R software package (http://r-project.org/; RRID:SCR_001905). All samples were processed as triplicates from three independent experiments. The MS proteomics data have been deposited to the ProteomeXchange Consortium via the PRIDE partner repository with the dataset identifier PXD064674 and 10.6019/PXD064674.

## Supplementary Materials

### This PDF file includes:

Figs. S1 to S11 Supplementary Text References
