# Supplementary material for: Steric repulsion counteracts ER–to–lipid droplet protein movement
Source: Sci Adv. 2025 Sep 24;11(39):eadu6998. doi: 10.1126/sciadv.adu6998 (PMC12459407; doi:10.1126/sciadv.adu6998)
Supplement: Supplementary file 1 — Figs. S1 to S11 Supplementary Text References [file sciadv.adu6998_sm.pdf]

Science Advances
AAAS

Supplementary Materials for

Steric repulsion counteracts ER–to–lipid droplet protein movement

Alicia Damm *et al.*

Corresponding author: Abdou Rachid Thiam, thiam@ens.fr

Sci. Adv. **11**, eadu6998 (2025)
DOI: 10.1126/sciadv.adu6998

This PDF file includes:

Figs. S1 to S11
Supplementary Text
References

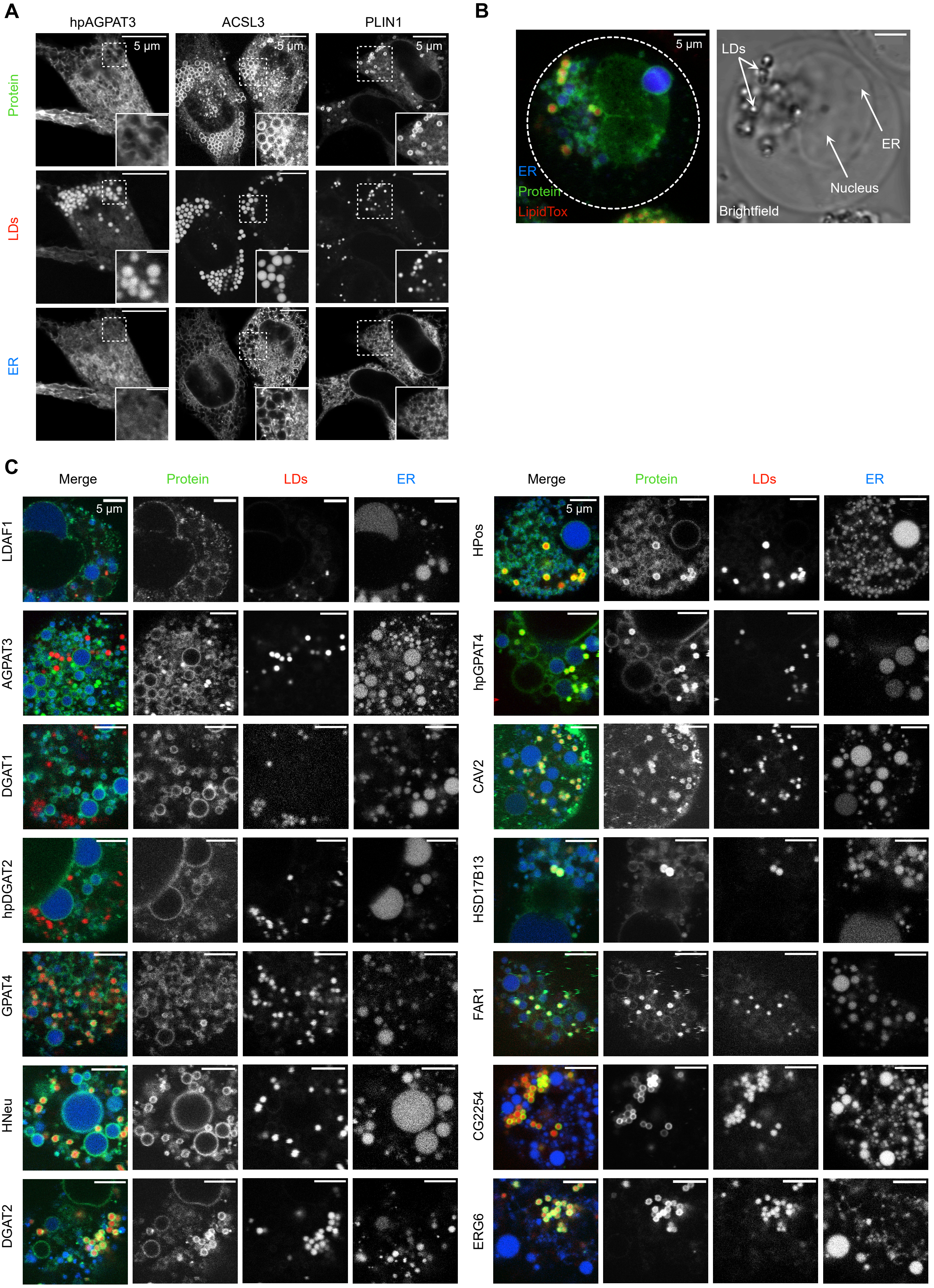

A
hpAGPAT3
ACSL3
PLIN1
Protein
LDs
ER
5 µm
B
ER
Protein
LipidTox
LDs
ER
Nucleus
Brightfield
C
Merge
Protein
LDs
ER
LDAF1
AGPAT3
DGAT1
hpDGAT2
GPAT4
HNeu
DGAT2
HPos
hpGPAT4
CAV2
HSD17B13
FAR1
CG2254
ERG6

Figure S1. Confocal images of transfected HeLa WT cells in normal or hypotonic media. (A) Separated channels of confocal microscopy images of cells in normal media, transfected with, respectively from left to right, hpAGPAT3, ACSL3, PLIN1. **(B)** Image of a cell in hypotonic media, fluorescence of ER lumen marker (blue), ER protein (green) and LDs marker (red) on the left and bright field on the right. **(C)** Confocal images of HeLa WT cells in hypotonic media for each protein. All scale bars are 5 µm.

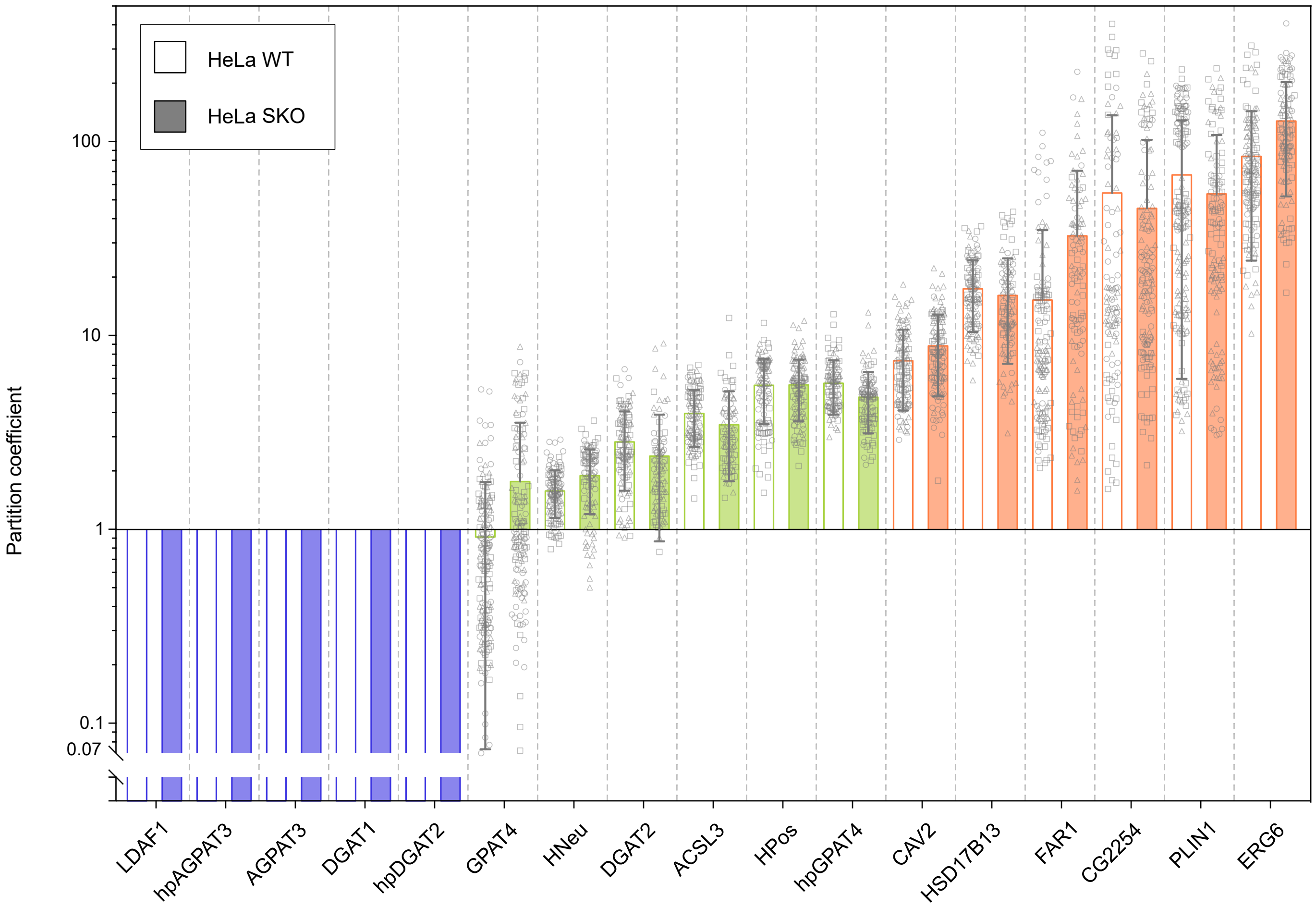
HeLa WT
HeLa SKO
Partition coefficient
100
10
1
0.1
0.07
LDAF1
hpAGPAT3
AGPAT3
DGAT1
hpDGAT2
GPAT4
HNeu
DGAT2
ACSL3
HPos
hpGPAT4
CAV2
HSD17B13
FAR1
CG2254
PLIN1
ERG6

Figure S2. Protein partition coefficient classification between HeLa WT and SKO cells. Partition coefficients for our 17 proteins subset are represented by mean value as a bar and standard deviation as gray whiskers (log scale), measured in HeLa WT (white bars) and in HeLa SKO (colored bars). All data points (= individual droplets) of three independent experiments are represented as circle, square and triangle gray symbols (between 3 to 5 cells for each experiment, with at least 8 droplets analyzed per cell).

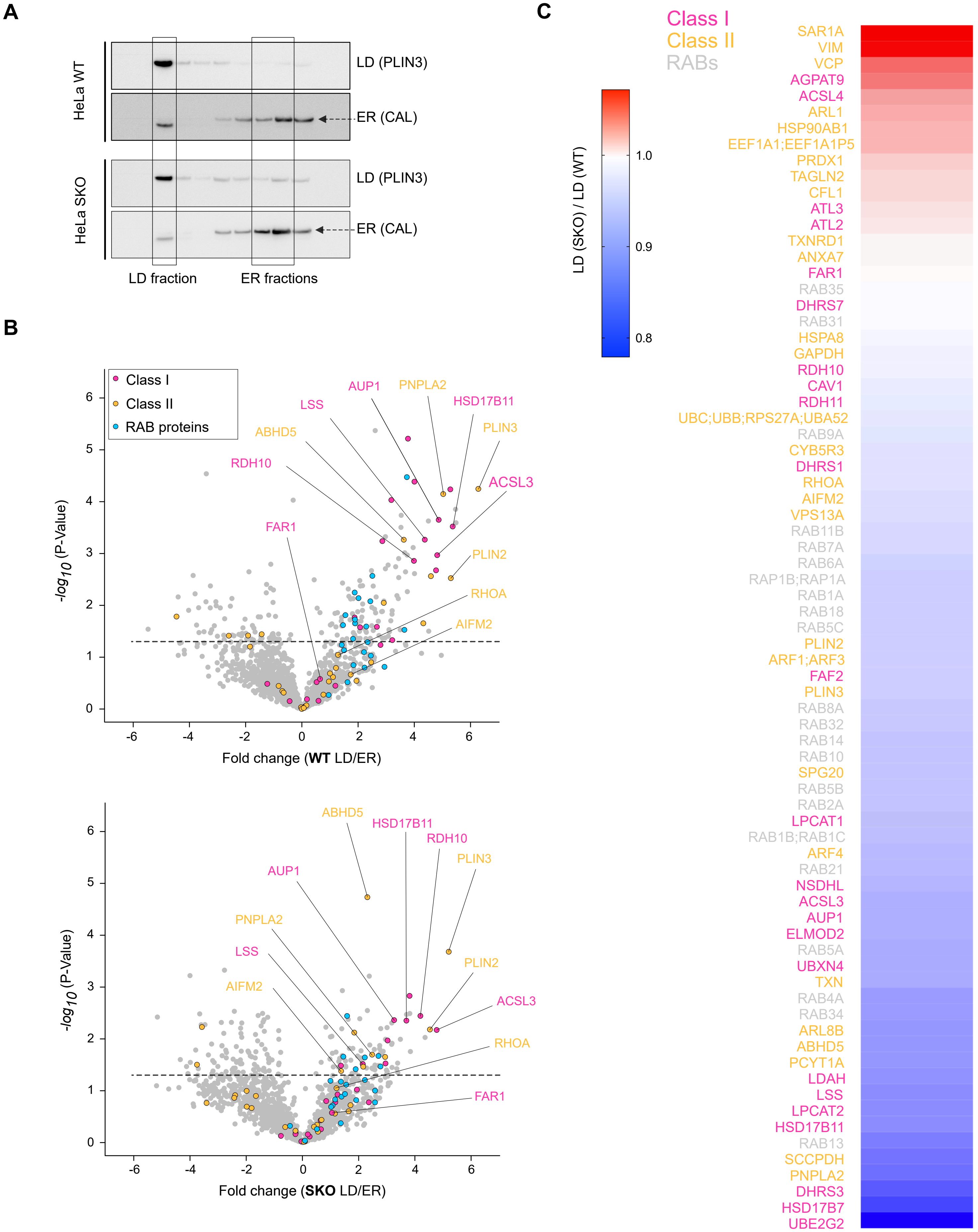
A
HeLa WT
HeLa SKO
LD (PLIN3)
ER (CAL)
LD (PLIN3)
ER (CAL)
LD fraction
ER fractions
B
Class I
Class II
RAB proteins
AUP1
PNPLA2
LSS
HSD17B11
PLIN3
ABHD5
RDH10
ACSL3
FAR1
PLIN2
RHOA
AIFM2
-log10 (P-Value)
Fold change (WT LD/ER)
ABHD5
HSD17B11
RDH10
PLIN3
AUP1
PNPLA2
LSS
PLIN2
AIFM2
ACSL3
RHOA
FAR1
Fold change (SKO LD/ER)
C
Class I
Class II
RABs
LD (SKO) / LD (WT)
1.0
0.9
0.8
SAR1A
VIM
VCP
AGPAT9
ACSL4
ARL1
HSP90AB1
EEF1A1;EEF1A1P5
PRDX1
TAGLN2
CFL1
ATL3
ATL2
TXNRD1
ANXA7
FAR1
RAB35
DHRS7
RAB31
HSPA8
GAPDH
RDH10
CAV1
RDH11
UBC;UBB;RPS27A;UBA52
RAB9A
CYB5R3
DHRS1
RHOA
AIFM2
VPS13A
RAB11B
RAB7A
RAB6A
RAP1B;RAP1A
RAB1A
RAB18
RAB5C
PLIN2
ARF1;ARF3
FAF2
PLIN3
RAB8A
RAB32
RAB14
RAB10
SPG20
RAB5B
RAB2A
LPCAT1
RAB1B;RAB1C
ARF4
RAB21
NSDHL
ACSL3
AUP1
ELMOD2
RAB5A
UBXN4
TXN
RAB4A
RAB34
ARL8B
ABHD5
PCYT1A
LDAH
LSS
LPCAT2
HSD17B11
RAB13
SCCPDH
PNPLA2
DHRS3
HSD17B7
UBE2G2

Figure S3. Proteomics analysis between HeLa WT and SKO cells. (A) Western Blots of cell lysates of HeLa WT and HeLa SKO separated by ultracentrifugation. First fraction corresponds to LDs (marked with PLIN3) and fractions six and seven were selected for ER fraction (marked by Calretinulin). **(B)** Relative abundance of proteins in LD fraction *versus* ER fraction analyzed by proteomics in HeLa WT (top) and HeLa SKO (bottom) as a volcano plot: statistical significance $-\log_{10}$(P-Value) as a function of the fold change of the relative LD abundance of class I (pink), class II (orange) and Rab family (blue) proteins. Proteins above the dashed line correspond to a P-Value < 0.05. **(C)** Heat map of the ratio of intensity of proteins in LD fraction between HeLa SKO and WT, analyzed by proteomics (80 relevant proteins were selected from class I in pink, class II in orange, and Rab family in grey).

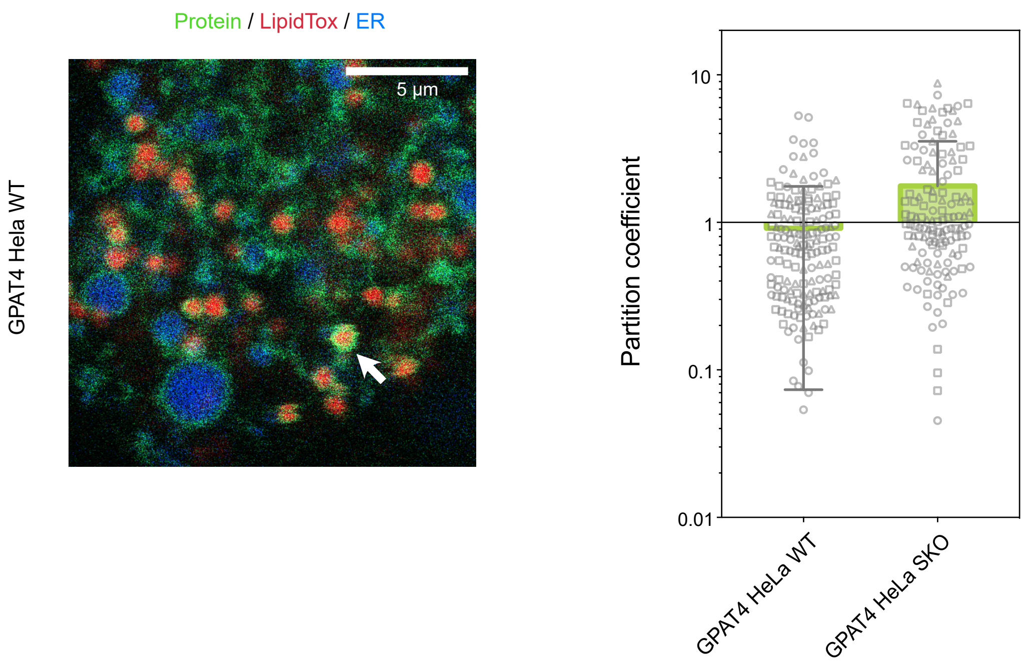
Protein / LipidTox / ER
GPAT4 Hela WT
5 µm
Partition coefficient
10
1
0.1
0.01
GPAT4 HeLa WT
GPAT4 HeLa SKO

Figure S4. The distinctive case of GPAT4. Left: Zoomed microscopy image of GPAT4 transfected HeLa WT in hypotonic media. White arrow points to LD with high targeting of GPAT4, other LDs have a lower protein intensity at their surface. Scale bar is 5 µm. Right: Individual droplet measurements of partition coefficient of GPAT4 in HeLa WT and SKO.

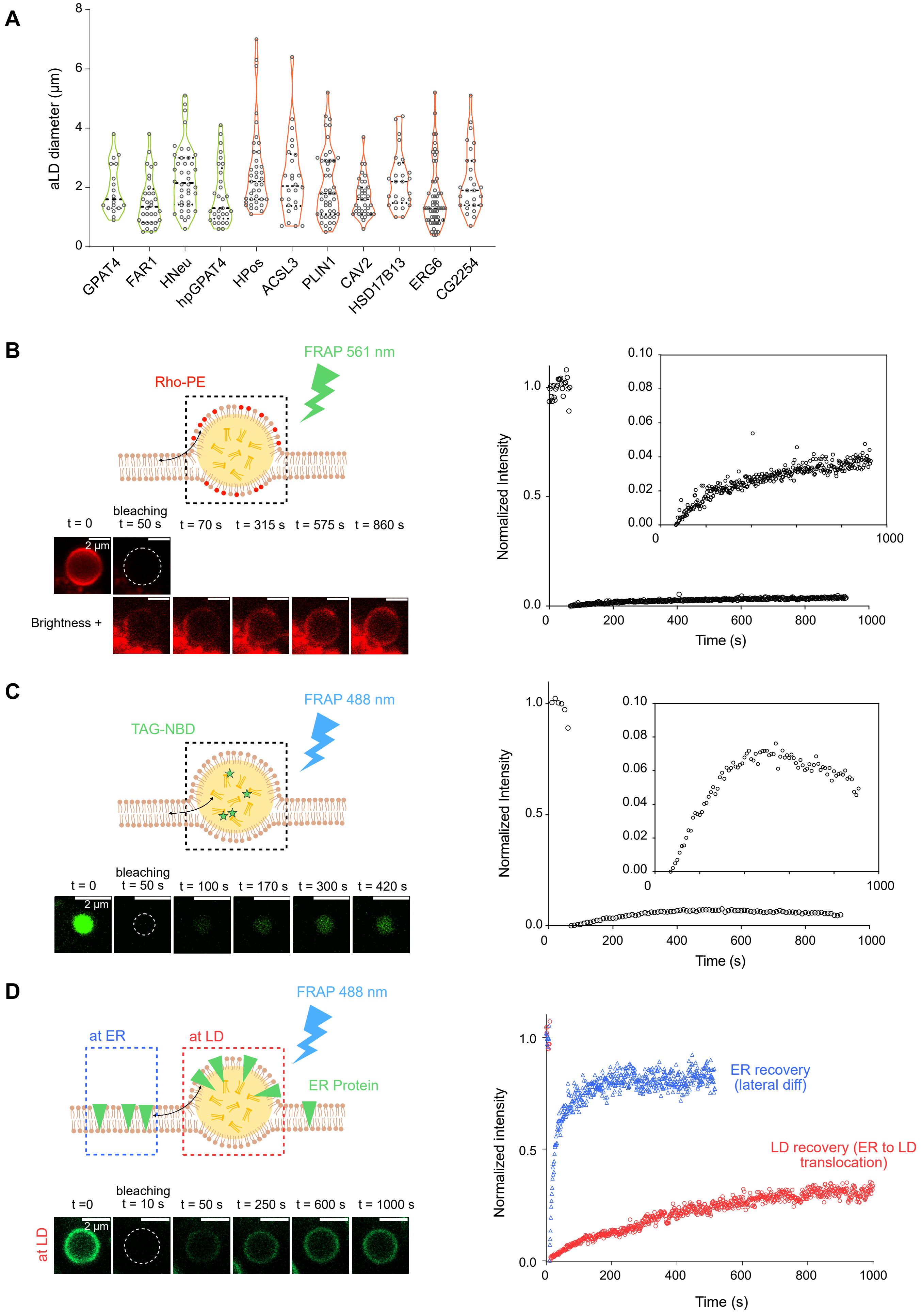
A
aLD diameter (µm)
GPAT4
FAR1
HNeu
hpGPAT4
HPos
ACSL3
PLIN1
CAV2
HSD17B13
ERG6
CG2254
B
FRAP 561 nm
Rho-PE
t = 0
bleaching
t = 50 s
t = 70 s
t = 315 s
t = 575 s
t = 860 s
2 µm
Brightness +
Normalized Intensity
Time (s)
C
FRAP 488 nm
TAG-NBD
t = 0
bleaching
t = 50 s
t = 100 s
t = 170 s
t = 300 s
t = 420 s
2 µm
Normalized Intensity
Time (s)
D
FRAP 488 nm
at ER
at LD
ER Protein
t =0
bleaching
t = 10 s
t = 50 s
t = 250 s
t = 600 s
t = 1000 s
at LD
2 µm
Normalized intensity
ER recovery
(lateral diff)
LD recovery (ER to LD
translocation)
Time (s)

Figure S5. Characterization of droplet size and their incorporation in GERVs by FRAP experiments. (A) Violin graphs of aLDs diameter in DEGERVs for the different proteins. **(B)** TAG-in-media emulsion was complemented with Rhodamine-DPPE (at 1:7000 w:w Rho-DPPE:TAG). We performed FRAP experiments by photobleaching the whole droplet and measuring the recovery of Rho-PE: graph shows normalized intensity at the droplet surface according to time, with a zoomed graph in the inset showing partial recovery of the PLs signal. **(C)** TAG-NBD-in-media emulsion was used and the whole TAG droplet was photobleached. The normalized volume intensity in the droplet according to time shows a partial recovery of the NLs signal (followed by a decay due to photobleaching at long time). **(D)** DEGERVs were prepared with HNeu-GFP transfected cells. We performed FRAP on the ER membrane, to measure lateral diffusion of the protein (blue), and by bleaching the whole aLD (red): a slower but consistent recovery was measured on LD as compared to ER, indicating that ER and LD can exchange protein, although limited by the reservoir capacity and the affinity of the protein for LD. Fluorescence recover shown has been corrected for photobleaching over time.

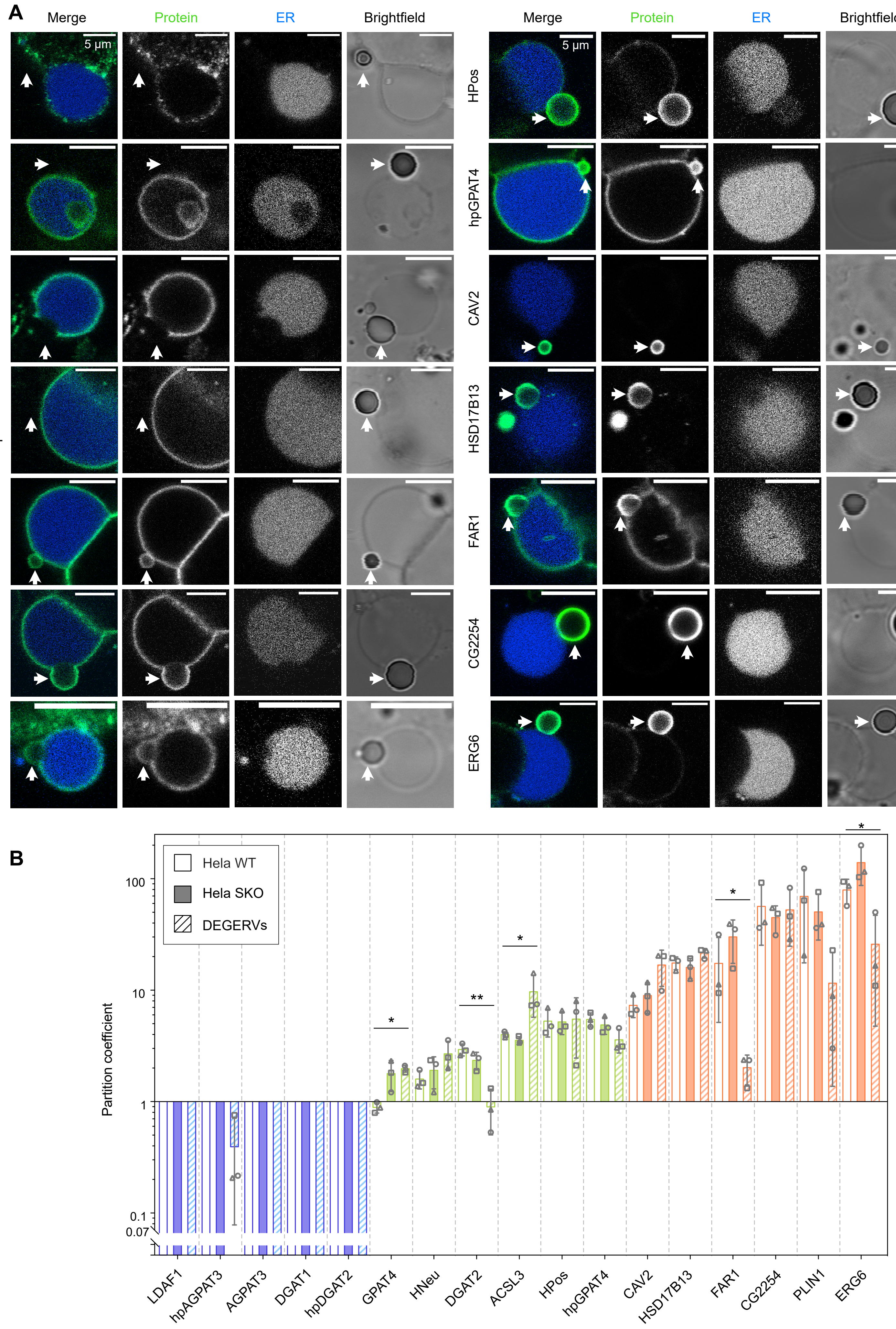
A
Merge
Protein
ER
Brightfield
5 µm
LDAF1
AGPAT3
DGAT1
hpDGAT2
GPAT4
HNeu
DGAT2
HPos
hpGPAT4
CAV2
HSD17B13
FAR1
CG2254
ERG6
B
Hela WT
Hela SKO
DEGERVs
Partition coefficient
100
10
1
0.1
0.07
LDAF1
hpAGPAT3
AGPAT3
DGAT1
hpDGAT2
GPAT4
HNeu
DGAT2
ACSL3
HPos
hpGPAT4
CAV2
HSD17B13
FAR1
CG2254
PLIN1
ERG6

Figure S6. Confocal images of DEGERVs and comparison of partition coefficients in cells (WT and SKO) and DEGERVs. (A) Confocal microscopy images for each protein with protein signal in green and ER lumen marker in blue. White arrows point to the GERV-embedded droplet. Scale bars are 5 µm. **(B)** Partition coefficients for our 17 proteins subset represented with mean value as a bar and standard deviation as gray whiskers (log scale), measured in HeLa WT (white bars), in HeLa SKO (colored bars), and in DEGERVs (striped bars). The average values of three independent experiments are represented as circle, square, and triangle gray symbols. Result of one-way ANOVA are shown on graph (see Table 2 for values), difference is non-significant when nothing is indicated.

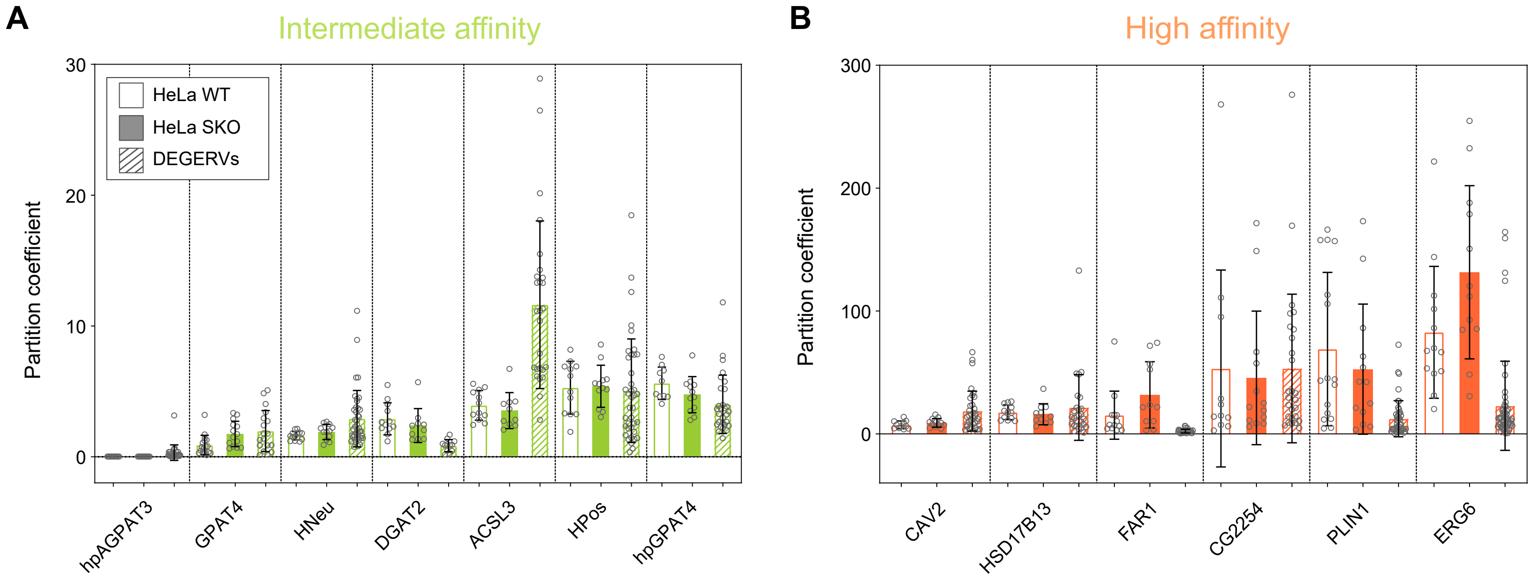

A
Intermediate affinity
HeLa WT
HeLa SKO
DEGERVs
Partition coefficient
30
20
10
0
hpAGPAT3
GPAT4
HNeu
DGAT2
ACSL3
HPos
hpGPAT4
B
High affinity
Partition coefficient
300
200
100
0
CAV2
HSD17B13
FAR1
CG2254
PLIN1
ERG6

Figure S7. Partition coefficients in cells (WT and SKO) and DEGERVs. (A,B) Partition coefficients for our 17 proteins subset represented with mean value as a bar and standard deviation as gray whiskers (log scale), measured in HeLa WT (white bars), in HeLa SKO (colored bars), and in DEGERVs (striped bars). Graphs are shown in linear scale, separated between intermediate affinity (A) and high affinity proteins (B). The average values measured per cell are represented as circle symbols. Note the different Y-axis limit.

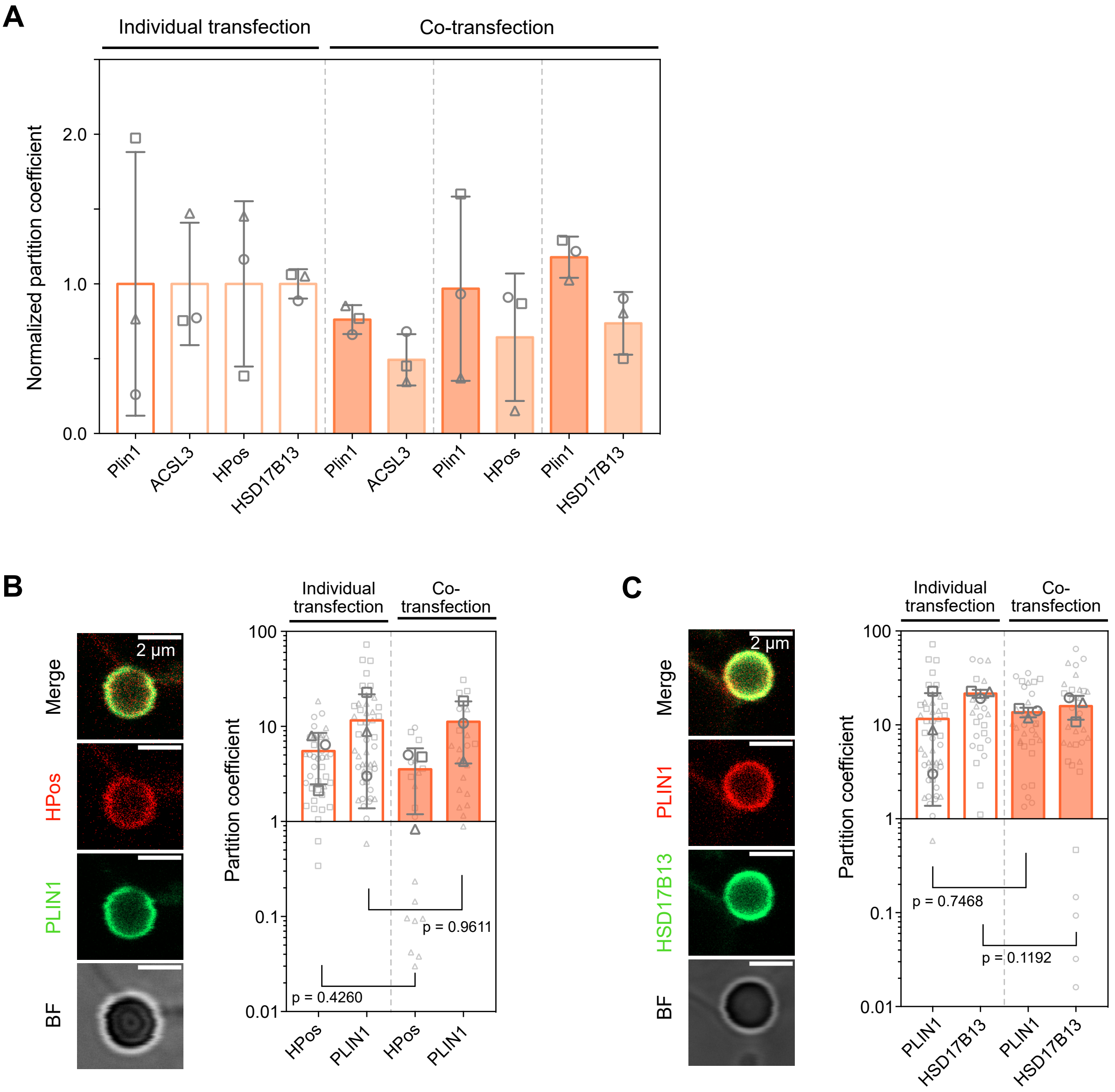
A
Individual transfection
Co-transfection
Normalized partition coefficient
2.0
1.0
0.0
Plin1
ACSL3
HPos
HSD17B13
Plin1
ACSL3
Plin1
HPos
Plin1
HSD17B13
B
2 µm
Merge
HPos
PLIN1
BF
Individual transfection
Co-transfection
Partition coefficient
100
10
1
0.1
0.01
p = 0.9611
p = 0.4260
HPos
PLIN1
HPos
PLIN1
C
2 µm
Merge
PLIN1
HSD17B13
BF
Individual transfection
Co-transfection
Partition coefficient
100
10
1
0.1
0.01
p = 0.7468
p = 0.1192
PLIN1
HSD17B13
PLIN1
HSD17B13

Figure S8. High-affinity proteins competition in DEGERVs. (A, B) We analyzed the competition between high-affinity proteins: PLIN1/ACSL3, PLIN1/HPos, and PLIN1/HSD17B13. **(A)** Partition coefficients are normalized by the average partitioning of a protein transfected individually (left part of graph). We can visualize that, even though other proteins are not fully displaced, PLIN1 dominates the relocation to LDs when competing with ACSL3, HPos or HSD17B13. **(B, C)** For each couple of proteins, we show confocal images of an embedded droplet (left panel) and we compare the partition coefficient of both proteins when transfected individually (right panel; white bars, Fig. 2F), and upon co-transfection (right panel; colored bars) for HPos/PLIN1 (B), and PLIN1/HSD17B13 (C). Average values of three independent experiments are represented as circle, square and triangle gray symbols. All data points are represented in light gray (between 3 to 22 droplets for each experiment). Results of the unpaired t-test are shown on the graph (see Tables 3 and 4).

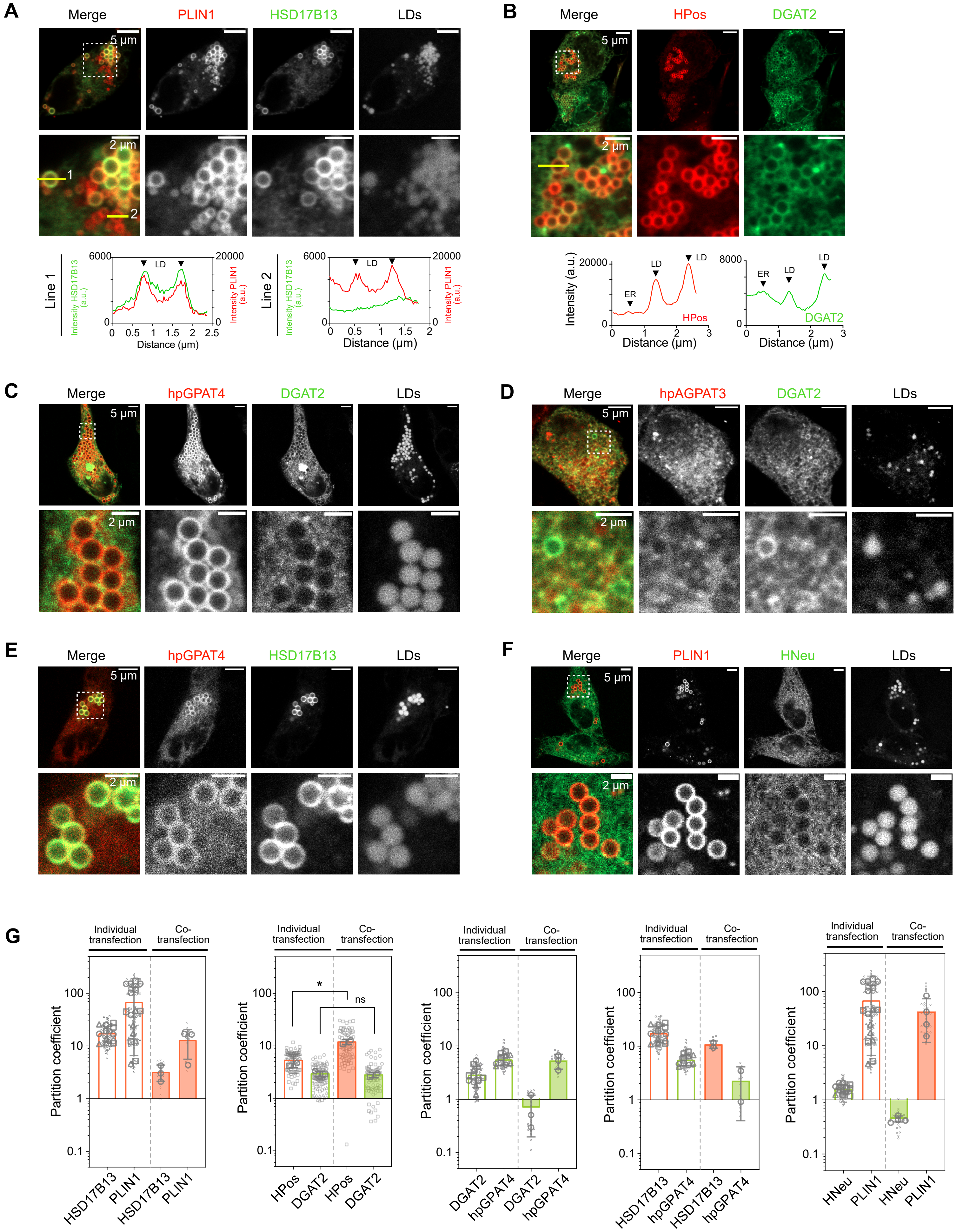
A
Merge
PLIN1
HSD17B13
LDs
5 µm
2 µm
1
2
Line 1
Intensity HSD17B13 (a.u.)
Intensity PLIN1 (a.u.)
Distance (µm)
LD
Line 2
B
Merge
HPos
DGAT2
Intensity (a.u.)
ER
LD
Distance (µm)
C
hpGPAT4
D
hpAGPAT3
E
F
HNeu
G
Individual transfection
Co-transfection
Partition coefficient
ns
*

Figure S9. High affinity proteins displace lower affinity proteins out of LDs in HeLa cells. (A, B, C, D, E, F) Confocal microscopy images of cells co-transfected with the following protein pairs: (A) PLIN1/HSD17B13, (B) HPos/DGAT2, (C) hpGPAT4/DGAT2, (D) hpAGPAT3/DGAT2, (E) hpGPAT4/HSD17B13 and (F) PLIN1/HNeu. A zoom of the square outlined region is provided on the lower row in each case. Line profiles in green and red channels along the yellow lines are shown in (A) and (B). **(G)** Quantification of the partition coefficients of both proteins when transfected individually (white bars, Fig. 1E), and upon co-transfection (colored bars). For all conditions: average values per cells from one replicate are shown for co-transfections (with individual LDs measurements shown in background). Except for HPos/DGAT2: average values of two independent experiments are represented as circle, square and triangle gray symbols (all data points are represented in light gray in the background). Result of unpaired t-test is shown on graph (see Tables 5 for detailed values).

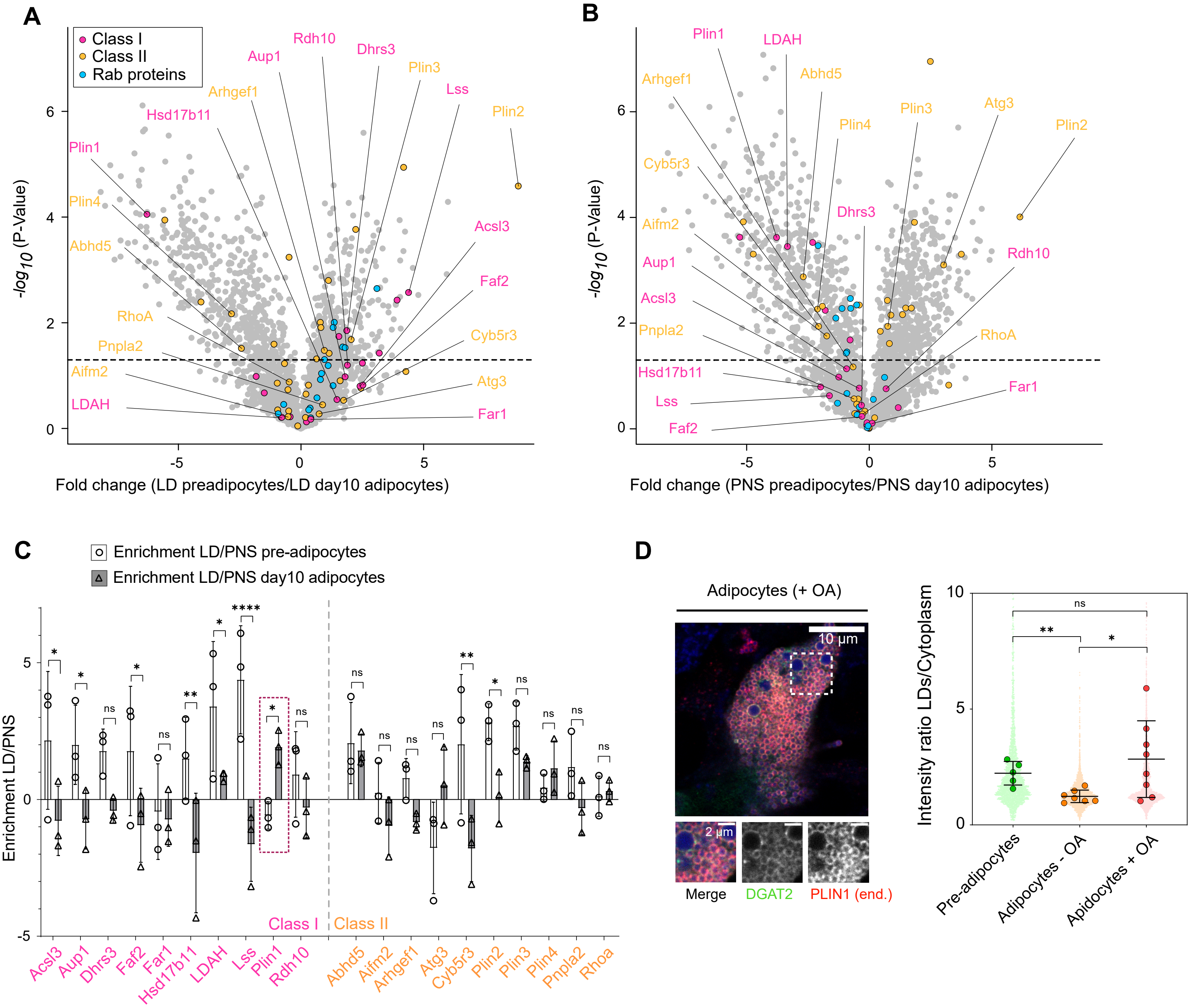
A
Class I
Class II
Rab proteins
-log10 (P-Value)
Fold change (LD preadipocytes/LD day10 adipocytes)
Plin1
Plin4
Abhd5
RhoA
Pnpla2
Aifm2
LDAH
Hsd17b11
Arhgef1
Aup1
Rdh10
Dhrs3
Plin3
Lss
Plin2
Acsl3
Faf2
Cyb5r3
Atg3
Far1
B
-log10 (P-Value)
Fold change (PNS preadipocytes/PNS day10 adipocytes)
Arhgef1
Cyb5r3
Aifm2
Aup1
Acsl3
Pnpla2
Hsd17b11
Lss
Faf2
Plin1
LDAH
Abhd5
Plin4
Dhrs3
Plin3
Atg3
Plin2
Rdh10
RhoA
Far1
C
Enrichment LD/PNS pre-adipocytes
Enrichment LD/PNS day10 adipocytes
Enrichment LD/PNS
Class I
Class II
Acsl3
Aup1
Dhrs3
Faf2
Far1
Hsd17b11
LDAH
Lss
Plin1
Rdh10
Abhd5
Aifm2
Arhgef1
Atg3
Cyb5r3
Plin2
Plin3
Plin4
Pnpla2
Rhoa
D
Adipocytes (+ OA)
10 µm
2 µm
Merge
DGAT2
PLIN1 (end.)
Intensity ratio LDs/Cytoplasm
Pre-adipocytes
Adipocytes - OA
Apidocytes + OA

Figure S10. Proteomics and immunofluorescence images of oleate-loaded pre-adipocytes and adipocytes. (A) Comparison of protein abundance in a fraction enriched for LDs prepared from preadipocytes treated with sodium oleate and from day 10 adipocytes. **(B)** As in (A) but for the post nuclear supernatants (PNS) which is the input material for LD enrichment used in (A). Statistical significance -log10(P-Value) is plotted over fold change. Class I proteins (pink), class II proteins (orange) and Rab family (blue) are highlighted. Proteins above the dashed line have a P-Value < 0.05. **(C)** Quantification of the enrichment in the LD-fraction over the input (postnuclear supernatant, PNS) in preadipocytes (circle, white bar) and in day10 adipocytes (triangle, grey bar). The data are expressed as mean of three independent replicates ± SD. Statistical significance was assessed in Prism using a multiple comparison test by the adaptive linear step of method of Benjamini, Krieger and Yekutieli. ns (p>0.1234), * (p>0.05), ** (p>0.005), *** (p>0.0002), **** (p<0.0001). ERTOLD (class I, pink) proteins are on the left, CYTOLD proteins on the right (class II, orange). **(D)** (Left) Confocal microscopy images of over-expressed DGAT2 (green) and endogenously expressed PLIN1 (red, by immunofluorescence) in adipocytes exposed to 200 μM OA overnight. LDs are labelled with LipidToxDeepRed (blue). (Right) Quantification of DGAT2 intensity at LDs, normalized by intensity in cytoplasm, in pre-adipocytes and adipocytes, without and with OA treatment. Average per cell, measured from two independent experiments, is shown as a large marker, individual LD values are shown in the background. The Y-axis limit to 10 prevents from displaying 50 individual data points that range to 20.

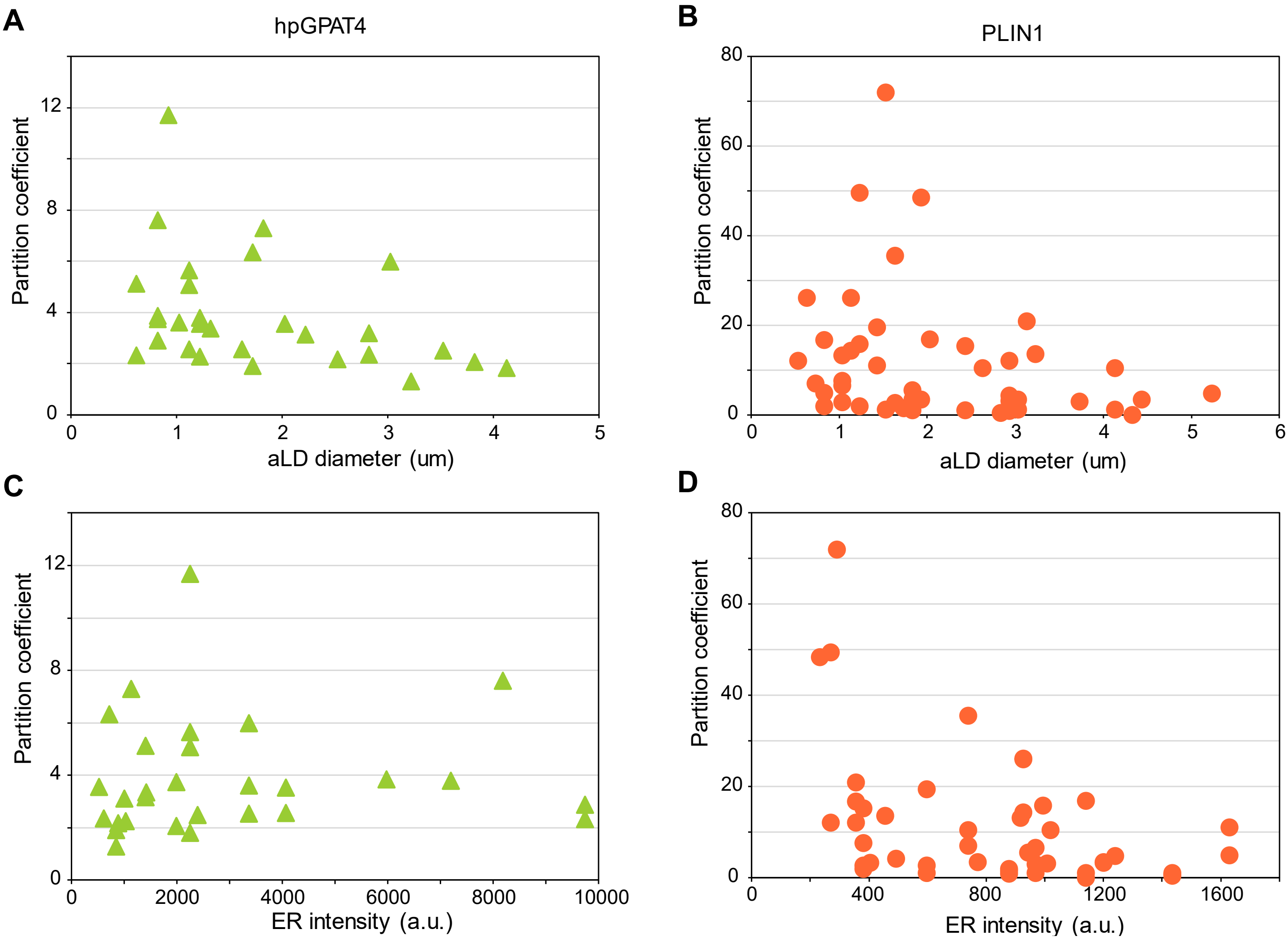
A
hpGPAT4
Partition coefficient
aLD diameter (um)
B
PLIN1
Partition coefficient
aLD diameter (um)
C
Partition coefficient
ER intensity (a.u.)
D
Partition coefficient
ER intensity (a.u.)

Figure S11. Effect of aLD diameter and protein's ER concentration on partition coefficients. (A, B) Partition coefficient plotted against aLD diameter for hpGPAT4 and PLIN1, respectively, an intermediate and a high affinity protein. **(C, D)** Partition coefficient plotted against protein's ER intensity (i.e. concentration of protein in the reservoir), for hpGPAT4 and PLIN1 respectively.

Supplementary Text.
Simple thermodynamic model of protein partition between Endoplasmic Reticulum and Lipid Droplet

We present a simple thermodynamic model of membrane protein partitioning between two subsystems. Our goal is not to make quantitative comparisons and tests with the *in vivo* experimental results, leading in particular to the extraction of parameter values. The complexity of the real system, the uncertainties in the measurements, and the unknown parameters of the model do not allow such comparisons. Our goal is rather to present and illustrate, through the use of a well-known model kept as simple as possible, the physical phenomena of steric exclusion and its influence on protein distribution. The model predictions with plausible values of the parameters are compared at a qualitative level with the tendencies observed experimentally.

1 Equilibrium condition

We consider a Lipid Droplet (LD) connected with the Endoplasmic Reticulum (ER). The continuity of the ER membrane leaflets with the LD interfaces allows some proteins to freely diffuse between the LD and the ER. At equilibrium, for each specy i that can be exchanged between the ER and LD, its distribution is imposed by the balance of the chemical potentials of that protein in the ER and in the LD,

$$\mu_i^{ER} = \mu_i^{LD} \ . \tag{S.1}$$

To express these chemical potentials in terms of the LD and ER physico-chemical parameters, and their protein compositions, we used a classical model of 2D fluid mixture taking into account the steric repulsion between protein molecules. This model is presented in the next section.

2 Model of membrane proteins mixture

The ER membrane and the LD surface covered with various proteins are both described using a classical model of 2D fluid mixture. In this model, the membrane of area A containing M protein species, is divided into N_0 sites of area a_0 (then $A = N_0 a_0$). Each site can be occupied by either 1 or 0 protein. The fact that a site cannot be occupied by more than one protein describes the effect of the steric repulsion between the proteins, also named "excluded area". The parameter a_0 accounts for the typical area occupied by a single protein and is taken to be equal for all protein species for simplicity. Other type of non-specific protein-protein interactions are neglected. We denote N_i the number of molecules of type i with $i \in \{1, 2, ..., M\}$, and $C_i = N_i a_0/A = N_i/N_0$, the surface fraction of proteins of type i. The total number of proteins N and total surface

fraction of proteins C are then,

$$N = \sum_{j=1}^{M} N_j \quad , \quad C = \sum_{j=1}^{M} C_j$$

The free energy of the protein mixture reads,

$$F = \sum_{i=1}^{M} \epsilon_i N_i - k_B T \left[N_0 \ln N_0 - (N_0 - N) \ln(N_0 - N) - \sum_{i=1}^{M} N_i \ln N_i \right] \ , \tag{S.2}$$

where ϵ_i is the free energy of a single protein i and accounts for the interactions between the protein and its host membrane, k_B is the Boltzmann constant and T is the temperature. The second term under bracket in (S.2) is the entropy of mixing of the proteins, including the effect of the limited accessible area. This free energy is widely used in the context of phase separation modelling, see for exemple (66). The version used here neglects the short range protein-protein interactions (except steric interactions). The chemical potential of a protein of type i is then,

$$\mu_i = \frac{\partial F}{\partial N_i} = \epsilon_i + k_B T \ln \left(\frac{C_i}{1 - C} \right) \tag{S.3}$$

Note that μ_i depends on the surface fraction of all the other species through the presence of $C = \sum_{j=1}^{M} C_j$ in the its expression.

For a given protein, its physical environment is not the same in the ER and in the LD. It follows that the parameter ϵ_i in (S.2) is not the same in the ER and in the LD. We denote ϵ_i^{ER} and ϵ_i^{LD}, their value in the ER and in a LD respectively. The surface fractions of proteins are also different in the ER and in the LD, and are denoted C_i^{ER} and C_i^{LD}.

3 Protein composition of the LD at equilibrium

The composition of the ER is regulated and its size is large as compared to a LD. The ER can thus be considered as a reservoir with fixed chemical potentials. Using the equilibrium condition (S.1), and equation (S.3) to express the chemical potential of the LD species, the equilibrium surface fraction of proteins i in the LD can be written,

$$C_i^{LD} = \frac{A_i}{1 + \sum_{j=1}^{M} A_j} \tag{S.4}$$

Each ER specy is thus characterized by a single fixed parameter A_i which reads,

$$A_i = \exp\left[(\mu_i^{ER} - \epsilon_i^{LD}) / k_B T \right]$$

This parameter corresponds to the surface fraction of protein i in the virtual situation where the steric interactions between proteins would be "switched off" *i.e.*, $C_i^{LD} = A_i$ in limit $a_0 \to 0$. The higher this parameter A_i, the higher the amount of protein i in the LD. One can notice from (S.4) that the density ratio of two species is,

$$\frac{C_i^{LD}}{C_j^{LD}} = \frac{A_i}{A_j} \ .$$

The denominator in equation (S.4) arises from the mutual competition between the proteins for the access to the LD surface : the higher the A_j with $j \neq i$, the lower the C_i^{LD}. Proteins of type j with a high affinity for the LD (*i.e.* large A_j) drastically reduces the amount of proteins i on the LD as compare to a situation where proteins j would be absent. As a conclusion, the amount of a given protein in the LD depends on the LD affinity of all the species, which mutually exclude each others.

4 Protein partitioning between the LD and ER

To relate the surface density of a protein in the LD to that in the ER, we specify the chemical potentials of the ER species. Starting from (S.3), the chemical potential of the protein i in the ER can be written,

$$\mu_i^{ER} = \tilde{\epsilon}_i^{ER} + k_B T \ln C_i^{ER} \ ,$$

where, $\tilde{\epsilon}_i^{ER} = \epsilon_i^{ER} - k_B T \ln(1 - C^{ER})$ is a free energy per protein which includes the steric interactions. In the ER, the total surface fraction of proteins C^{ER} is not low but the surface fraction of each specy i is low, $C_i^{ER} \ll 1$. It follows that the parameter $\tilde{\epsilon}_i^{ER}$ can be taken as a constant independent of C_i^{ER}. We can then write,

$$A_i = K_i C_i^{ER} \ , \tag{S.5}$$

where the parameter,

$$K_i = \exp\left[(\tilde{\epsilon}_i^{ER} - \epsilon_i^{LD})/k_B T\right]$$

characterizes the relative affinity of a ER protein of type i for the LD. If $\epsilon_i^{LD} > \tilde{\epsilon}_i^{ER}$ then $K_i > 1$, the proteins i prefer to be on the LD surface rather than on the ER membrane and conversely. Note that the energies $\epsilon_i^{ER,LD}$ should be of the order of several $k_B T$. The parameter K_i can thus be possibly as large as a few hundreds.

5 Competition between two protein species

To draw comparisons with the experimental results of the paper, we applied equation (S.4) to the case where two ER proteins species are tagged and are indexed 1 and 2. Of course the ER contains many other untagged protein species. The equilibrium density of the tagged proteins in the LD reads (S.4),

$$C_1^{LD} = \frac{A_1}{1 + A_1 + A_2 + A_{ER}} \quad \text{and} \quad C_2^{LD} = \frac{A_2}{1 + A_1 + A_2 + A_{ER}} \quad \text{with} \quad A_{ER} = \sum_{j=3}^{M} A_j \ . \tag{S.6}$$

The effect of all the other untagged proteins is thus characterized by a single parameter A_{ER}. In other word, this pool of untagged proteins has the same effect as a single third protein type indexed 3, $A_{ER} = A_3 = K_3 C_3^{ER}$, where in the second equality C_3^{ER} is the total surface fraction of untagged proteins and K_3 the average of value K_j over these proteins.

Case of a binary mixture.
To understand the steric exclusion effect, it is first interesting to consider the fictive situation with only two proteins (no untagged proteins $A_{ER} = 0$) and compare the case with protein 1 alone ($A_2 = 0$) and both protein present. In the second case, the amount of protein 1 in the LD will be reduced by a factor $(1 + A_1)/(1 + A_1 + A_2)$ as compared to the first case. This reduction is significant if A_2 is (i) close or larger than 1, which implies a high affinity of protein 2 for the LD, and (ii) of the order or larger than A_1. See the histograms of Figure 3E of the main part, where we used $A_1 = 2.5$ and $A_2 = 15$. To illustrate the exclusion of the protein 1 by the protein 2, we also show in the figure 3E the evolution of C_1^{LD} and C_2^{LD} when the density of protein 2 in the ER, C_2^{ER}, is increased little by little, thereby increasing $A_2 = K_2 C_2^{ER}$. We used $K_2 = 300$ and $C_1^{ER} = C_2^{ER} = 0.05$.

Effect of the pool of untagged proteins.
The competition between two proteins discussed above is reduced in presence of the other ER proteins willing to go the LD and contributing to the parameter A_{ER} in (S.6). According to equation (S.6), the larger this parameter A_{ER} the lower the direct effect of A_2 on C_1^{LD} (and of

A_1 on C_2^{LD}). Revisiting the comparison of the preceding paragraph (protein 2 absent *vs* protein 2 present), the reduction factor of the density of protein 1 in the LD due to the presence of protein 2 is now $(1 + A_1 + A_{ER})/(1 + A_1 + A_2 + A_{ER})$. This factor deviates significantly from 1 only if A_2 is large or comparable to A_{ER}.

Comparison with experiments.
In the *in vivo* experiments, the ER membrane always contains the full set of proteins. The effect of the transfection is to increase by an (unknown) factor the density of the transfected proteins in the ER, thereby increasing its propensity to go the the LD, equationS.5). In the model, it can be mimicked by increasing C_i^{ER} and thus A_i with i the label of the transfected protein, by an arbitrary factor. We did so for three pairs of tagged proteins with different couples of values (A_1, A_2), see Figure 3G.

REFERENCES AND NOTES

1. M. Bosch, M. J. Sweet, R. G. Parton, A. Pol, Lipid droplets and the host–pathogen dynamic: FATal attraction? *J. Cell Biol.* **220**, e202104005 (2021).

2. M. A. Welte, A. P. Gould, Lipid droplet functions beyond energy storage. *Biochim. Biophys. Acta Mol. Cell Biol. Lipids* **1862**, 1260–1272 (2017).

3. J. A. Olzmann, P. Carvalho, Dynamics and functions of lipid droplets. *Nat. Rev. Mol. Cell Biol.* **20**, 137–155 (2019).

4. T. C. Walther, R. V. Farese, Lipid droplets and cellular lipid metabolism. *Annu. Rev. Biochem.* **81**, 687–714 (2012).

5. A. R. Thiam, L. Forêt, The physics of lipid droplet nucleation, growth and budding. *Biochim. Biophys. Acta* **1861**, 715–722 (2016).

6. A. Chorlay, L. Monticelli, J. V. Ferreira, K. B. M'barek, D. Ajjaji, S. Wang, E. Johnson, R. Beck, M. Omrane, M. Beller, Membrane asymmetry imposes directionality on lipid droplet emergence from the ER. *Dev. Cell* **50**, 25–42.e7 (2019).

7. A. R. Thiam, R. V. Farese Jr., T. C. Walther, The biophysics and cell biology of lipid droplets. *Nat. Rev. Mol. Cell Biol.* **14**, 775–786 (2013).

8. K. B. M'barek, D. Ajjaji, A. Chorlay, S. Vanni, L. Forêt, A. R. Thiam, ER membrane phospholipids and surface tension control cellular lipid droplet formation. *Dev. Cell* **41**, 591–604.e7 (2017).

9. A. Chorlay, A. R. Thiam, Neutral lipids regulate amphipathic helix affinity for model lipid droplets. *J. Cell Biol.* **219**, e201907099 (2020).

10. A. R. Thiam, I. Dugail, Lipid droplet–membrane contact sites–from protein binding to function. *J. Cell Sci.* **132**, jcs230169 (2019).

11. K. Bersuker, J. A. Olzmann, Establishing the lipid droplet proteome: Mechanisms of lipid droplet protein targeting and degradation. *Biochim. Biophys. Acta Mol. Cell Biol. Lipids* **1862**, 1166–1177 (2017).

12. N. Kory, R. V. Farese Jr., T. C. Walther, Targeting fat: Mechanisms of protein localization to lipid droplets. *Trends Cell Biol.* **26**, 535–546 (2016).

13. M.-J. Olarte, J. M. Swanson, T. C. Walther, R. V. Farese Jr., The CYTOLD and ERTOLD pathways for lipid droplet–protein targeting. *Trends Biochem. Sci.* **47**, 39–51 (2022).

14. A. Čopič, S. Antoine-Bally, M. Gimenez-Andres, C. T. Garay, B. Antonny, M. M. Manni, S. Pagnotta, J. Guihot, C. L. Jackson, A giant amphipathic helix from a perilipin that is adapted for coating lipid droplets. *Nat. Commun.* **9**, 1332 (2018).

15. C. Prévost, M. E. Sharp, N. Kory, Q. Lin, G. A. Voth, R. V. Farese Jr., T. C. Walther, Mechanism and determinants of amphipathic helix-containing protein targeting to lipid droplets. *Dev. Cell* **44**, 73–86.e4 (2018).

16. N. Kory, A.-R. Thiam, R. V. Farese Jr., T. C. Walther, Protein crowding is a determinant of lipid droplet protein composition. *Dev. Cell* **34**, 351–363 (2015).

17. L. Caillon, V. Nieto, P. Gehan, M. Omrane, N. Rodriguez, L. Monticelli, A. R. Thiam, Triacylglycerols sequester monotopic membrane proteins to lipid droplets. *Nat. Commun.* **11**, 3944 (2020).

18. R. Dhiman, R. S. Perera, C. S. Poojari, H. T. Wiedemann, R. Kappl, C. W. Kay, J. S. Hub, B. Schrul, Hairpin protein partitioning from the ER to lipid droplets involves major structural rearrangements. *Nat. Commun.* **15**, 4504 (2024).

19. N. Jacquier, V. Choudhary, M. Mari, A. Toulmay, F. Reggiori, R. Schneiter, Lipid droplets are functionally connected to the endoplasmic reticulum in Saccharomyces cerevisiae. *J. Cell Sci.* **124**, 2424–2437 (2011).

20. D. F. Markgraf, R. W. Klemm, M. Junker, H. K. Hannibal-Bach, C. S. Ejsing, T. A. Rapoport, An ER protein functionally couples neutral lipid metabolism on lipid droplets to membrane lipid synthesis in the ER. *Cell Rep.* **6**, 44–55 (2014).

21. M.-J. Olarte, S. Kim, M. E. Sharp, J. M. Swanson, R. V. Farese Jr., T. C. Walther, Determinants of endoplasmic reticulum-to-lipid droplet protein targeting. *Dev. Cell* **54**, 471–487.e7 (2020).

22. A. Kassan, A. Herms, A. Fernández-Vidal, M. Bosch, N. L. Schieber, B. J. Reddy, A. Fajardo, M. Gelabert-Baldrich, F. Tebar, C. Enrich, Acyl-CoA synthetase 3 promotes lipid droplet biogenesis in ER microdomains. *J. Cell Biol.* **203**, 985–1001 (2013).

23. P. J. McFie, S. L. Banman, S. J. Stone, Diacylglycerol acyltransferase-2 contains a C-terminal sequence that interacts with lipid droplets. *Biochim. Biophys. Acta Mol. Cell Biol. Lipids* **1863**, 1068–1081 (2018).

24. T. Fujimoto, H. Kogo, K. Ishiguro, K. Tauchi, R. Nomura, Caveolin-2 is targeted to lipid droplets, a new "membrane domain" in the cell. *J. Cell Biol.* **152**, 1079–1086 (2001).

25. F. Morales-Paytuví, A. Fajardo, C. Ruiz-Mirapeix, J. Rae, F. Tebar, M. Bosch, C. Enrich, B. M. Collins, R. G. Parton, A. Pol, Early proteostasis of caveolins synchronizes trafficking, degradation, and oligomerization to prevent toxic aggregation. *J. Cell Biol.* **222**, e202204020 (2023).

26. M. Carpentier, M. Omrane, J. Trager, M. Zouiouich, R. Shaaban, X. Prieur, M. Palard, N. El Khallouki, F. Giordano, T. Harayama, Seipin regulates caveolin-1 trafficking and organelle crosstalk. bioRxiv2024.09.17.613438 [Preprint] (2024). https://doi.org/10.1101/2024.09.17.613438.

27. A. Mizrak, J. Kaestel-Hansen, J. Matthias, J. W. Harper, N. S. Hatzakis, T. C. Walther, R. V. Farese Jr., Single-molecule analysis of protein targeting from the endoplasmic reticulum to lipid droplets. bioRxiv 2024.08.27.610018 [Preprint] (2024). https://doi.org/10.1101/2024.08.27.610018.

28. J. Song, A. Mizrak, C.-W. Lee, M. Cicconet, Z. W. Lai, W.-C. Tang, C.-H. Lu, S. E. Mohr, R. V. Farese Jr., T. C. Walther, Identification of two pathways mediating protein targeting from ER to lipid droplets. *Nat. Cell Biol.* **24**, 1364–1377 (2022).

29. M. Majchrzak, O. Stojanović, D. Ajjaji, K. B. M'barek, M. Omrane, A. R. Thiam, R. W. Klemm, Perilipin membrane integration determines lipid droplet heterogeneity in differentiating adipocytes. *Cell Rep.* **43**, 114093 (2024).

30. Y. A. Klug, J. C. Deme, R. A. Corey, M. F. Renne, P. J. Stansfeld, S. M. Lea, P. Carvalho, Mechanism of lipid droplet formation by the yeast Sei1/Ldb16 Seipin complex. *Nat. Commun.* **12**, 5892 (2021).

31. X. Sui, H. Arlt, K. P. Brock, Z. W. Lai, F. DiMaio, D. S. Marks, M. Liao, R. V. Farese, T. C. Walther, Cryo–electron microscopy structure of the lipid droplet–formation protein seipin. *J. Cell Biol.* **217**, 4080–4091 (2018).

32. W.-C. Su, Y.-H. Lin, M. Pagac, C.-W. Wang, Seipin negatively regulates sphingolipid production at the ER–LD contact site. *J. Cell Biol.* **218**, 3663–3680 (2019).

33. J. Chung, X. Wu, T. J. Lambert, Z. W. Lai, T. C. Walther, R. V. Farese Jr., LDAF1 and seipin form a lipid droplet assembly complex. *Dev. Cell* **51**, 551–563.e7 (2019).

34. I. G. Castro, M. Eisenberg-Bord, E. Persiani, J. J. Rochford, M. Schuldiner, M. Bohnert, Promethin is a conserved seipin partner protein. *Cells* **8**, 268 (2019).

35. V. T. Salo, I. Belevich, S. Li, L. Karhinen, H. Vihinen, C. Vigouroux, J. Magré, C. Thiele, M. Hölttä-Vuori, E. Jokitalo, Seipin regulates ER–lipid droplet contacts and cargo delivery. *EMBO J.* **35**, 2699–2716 (2016).

36. F. Wilfling, H. Wang, J. T. Haas, N. Krahmer, T. J. Gould, A. Uchida, J.-X. Cheng, M. Graham, R. Christiano, F. Fröhlich, Triacylglycerol synthesis enzymes mediate lipid droplet growth by relocalizing from the ER to lipid droplets. *Dev. Cell* **24**, 384–399 (2013).

37. F. Wilfling, A. R. Thiam, M.-J. Olarte, J. Wang, R. Beck, T. J. Gould, E. S. Allgeyer, F. Pincet, J. Bewersdorf, R. V. Farese Jr., T. C. Walther, Arf1/COPI machinery acts directly on

lipid droplets and enables their connection to the ER for protein targeting. *Elife* **3**, e01607 (2014).

38. H. Wang, M. Becuwe, B. E. Housden, C. Chitraju, A. J. Porras, M. M. Graham, X. N. Liu, A. R. Thiam, D. B. Savage, A. K. Agarwal, A. Garg, M. J. Olarte, Q. Lin, F. Fröhlich, H. K. Hannibal-Bach, S. Upadhyayula, N. Perrimon, T. Kirchhausen, C. S. Ejsing, T. C. Walther, R. V. Farese Jr., Seipin is required for converting nascent to mature lipid droplets. *Elife* **5**, e16582 (2016).

39. R. Dhiman, S. Caesar, A. R. Thiam, B. Schrul, Mechanisms of protein targeting to lipid droplets: A unified cell biological and biophysical perspective. *Semin. Cell Dev. Biol.* **108**, 4–13 (2020).

40. A. R. Thiam, B. Antonny, J. Wang, J. Delacotte, F. Wilfling, T. C. Walther, R. Beck, J. E. Rothman, F. Pincet, COPI buds 60-nm lipid droplets from reconstituted water–phospholipid–triacylglyceride interfaces, suggesting a tension clamp function. *Proc. Natl. Acad. Sci. U.S.A.* **110**, 13244–13249 (2013).

41. A. Chorlay, L. Forêt, A. R. Thiam, Origin of gradients in lipid density and surface tension between connected lipid droplet and bilayer. *Biophys. J.* **120**, 5491–5503 (2021).

42. S. Rogers, L. Gui, A. Kovalenko, V. Zoni, M. Carpentier, K. Ramji, K. Ben Mbarek, A. Bacle, P. Fuchs, P. Campomanes, E. Reetz, N. O. Speer, E. Reynolds, A. R. Thiam, S. Vanni, D. Nicastro, W. M. Henne, Triglyceride lipolysis triggers liquid crystalline phases in lipid droplets and alters the LD proteome. *J. Cell Biol.* **221**, e202205053 (2022).

43. D. Ajjaji, K. B. M'barek, B. Boson, M. Omrane, A. Gassama-Diagne, M. Blaud, F. Penin, E. Diaz, B. Ducos, F.-L. Cosset, A. R. Thiam, Hepatitis C virus core protein uses triacylglycerols to fold onto the endoplasmic reticulum membrane. *Traffic* **23**, 63–80 (2022).

44. D. Ajjaji, K. B. M'barek, M. L. Mimmack, C. England, H. Herscovitz, L. Dong, R. G. Kay, S. Patel, V. Saudek, D. M. Small, Dual binding motifs underpin the hierarchical association of perilipins1–3 with lipid droplets. *Mol. Biol. Cell* **30**, 703–716 (2019).

45. A. Santinho, M. Carpentier, J. Lopes Sampaio, M. Omrane, A. R. Thiam, Giant organelle vesicles to uncover intracellular membrane mechanics and plasticity. *Nat. Commun.* **15**, 3767 (2024).

46. A. Grippa, L. Buxó, G. Mora, C. Funaya, F.-Z. Idrissi, F. Mancuso, R. Gomez, J. Muntanyà, E. Sabidó, P. Carvalho, The seipin complex Fld1/Ldb16 stabilizes ER–lipid droplet contact sites. *J. Cell Biol.* **211**, 829–844 (2015).

47. W. Fei, L. Zhong, M. T. Ta, G. Shui, M. R. Wenk, H. Yang, The size and phospholipid composition of lipid droplets can influence their proteome. *Biochem. Biophys. Res. Commun.* **415**, 455–462 (2011).

48. A. Chorlay, A. Santinho, A. R. Thiam, Making droplet-embedded vesicles to model cellular lipid droplets. *STAR Protoc.* **1**, 100116 (2020).

49. A. Chorlay, A. R. Thiam, An asymmetry in monolayer tension regulates lipid droplet budding direction. *Biophys. J.* **114**, 631–640 (2018).

50. N. E. Wolins, D. L. Brasaemle, P. E. Bickel, A proposed model of fat packaging by exchangeable lipid droplet proteins. *FEBS Lett.* **580**, 5484–5491 (2006).

51. Z. Sun, J. Gong, H. Wu, W. Xu, L. Wu, D. Xu, J. Gao, J. Wu, H. Yang, M. Yang, P. Li, Perilipin1 promotes unilocular lipid droplet formation through the activation of Fsp27 in adipocytes. *Nat. Commun.* **4**, 1594 (2013).

52. L. Xu, L. Li, L. Wu, P. Li, F. Chen, CIDE proteins and their regulatory mechanisms in lipid droplet fusion and growth. *FEBS Lett.* **598**, 1154–1169 (2024).

53. K. G. Soni, G. A. Mardones, R. Sougrat, E. Smirnova, C. L. Jackson, J. S. Bonifacino, Coatomer-dependent protein delivery to lipid droplets. *J. Cell Sci.* **122**, 1834–1841 (2009).

54. A. S. Greenberg, J. J. Egan, S. A. Wek, N. B. Garty, E. J. Blanchette-Mackie, C. Londos, Perilipin, a major hormonally regulated adipocyte-specific phosphoprotein associated with the periphery of lipid storage droplets. *J. Biol. Chem.* **266**, 11341–11346 (1991).

55. C. Sztalryd, D. L. Brasaemle, The perilipin family of lipid droplet proteins: Gatekeepers of intracellular lipolysis. *Biochim. Biophys. Acta Mol. Cell Biol. Lipids* **1862**, 1221–1232 (2017).

56. H. Miyoshi, S. C. Souza, H.-H. Zhang, K. J. Strissel, M. A. Christoffolete, J. Kovsan, A. Rudich, F. B. Kraemer, A. C. Bianco, M. S. Obin, Perilipin promotes hormone-sensitive lipase-mediated adipocyte lipolysis via phosphorylation-dependent and-independent mechanisms. *J. Biol. Chem.* **281**, 15837–15844 (2006).

57. U. Schmidt, M. Weigert, C. Broaddus, G. Myers, "Cell detection with star-convex polygons" in *Medical Image Computing and Computer Assisted Intervention–MICCAI 2018*, vol. 11071 of *Lecture Notes in Computer Science*, A. F. Frangi, J. A. Schnabel, C. Davatzikos, C. Alberola-López, G. Fichtinger, Eds. (Springer International Publishing, 2018), pp. 265–273.

58. C. Stringer, T. Wang, M. Michaelos, M. Pachitariu, Cellpose: A generalist algorithm for cellular segmentation. *Nat. Methods* **18**, 100–106 (2021).

59. A. E. Carpenter, T. R. Jones, M. R. Lamprecht, C. Clarke, I. H. Kang, O. Friman, D. A. Guertin, J. H. Chang, R. A. Lindquist, J. Moffat, P. Golland, D. M. Sabatini, CellProfiler: Image analysis software for identifying and quantifying cell phenotypes. *Genome Biol.* **7**, R100 (2006).

60. J. V. Olsen, B. Macek, O. Lange, A. Makarov, S. Horning, M. Mann, Higher-energy C-trap dissociation for peptide modification analysis. *Nat. Methods* **4**, 709–712 (2007).

61. J. Cox, M. Mann, MaxQuant enables high peptide identification rates, individualized ppb-range mass accuracies and proteome-wide protein quantification. *Nat. Biotechnol.* **26**, 1367–1372 (2008).

62. J. Cox, M. Mann, Quantitative, high-resolution proteomics for data-driven systems biology. *Annu. Rev. Biochem.* **80**, 273–299 (2011).

63. Y. Perez-Riverol, C. Bandla, D. J. Kundu, S. Kamatchinathan, J. Bai, S. Hewapathirana, N. S. John, A. Prakash, M. Walzer, S. Wang, The PRIDE database at 20 years: 2025 update. *Nucleic Acids Res.* **53**, D543–D553 (2025).

64. N. C. Hubner, A. W. Bird, J. Cox, B. Splettstoesser, P. Bandilla, I. Poser, A. Hyman, M. Mann, Quantitative proteomics combined with BAC TransgeneOmics reveals in vivo protein interactions. *J. Cell Biol.* **189**, 739–754 (2010).

65. S. Eising, L. Thiele, F. Fröhlich, A systematic approach to identify recycling endocytic cargo depending on the GARP complex. *Elife* **8**, e42837 (2019).

66. J. Berry, C. P. Brangwynne, M. Haataja, Physical principles of intracellular organization via active and passive phase transitions. *Rep. Prog. Phys.* **81**, 046601 (2018).
